# Maternal exposure to diluted diesel engine exhaust alters placental function and induces intergenerational effects in rabbits

**DOI:** 10.1186/s12989-016-0151-7

**Published:** 2016-07-26

**Authors:** Sarah A. Valentino, Anne Tarrade, Josiane Aioun, Eve Mourier, Christophe Richard, Michèle Dahirel, Delphine Rousseau-Ralliard, Natalie Fournier, Marie-Christine Aubrière, Marie-Sylvie Lallemand, Sylvaine Camous, Marine Guinot, Madia Charlier, Etienne Aujean, Hala Al Adhami, Paul H. Fokkens, Lydiane Agier, John A. Boere, Flemming R. Cassee, Rémy Slama, Pascale Chavatte-Palmer

**Affiliations:** 1 UMR BDR, INRA, ENVA, Université Paris Saclay, 78350 Jouy en Josas, France; 2 PremUp Foundation, Paris, France; 3 UFR de Pharmacie, Univ Paris-Sud, EA 4041/4529 Lip (Sys), Châtenay-Malabry, France; 4 Hôpital Européen Georges Pompidou (AP-HP), Laboratoire de Biochimie, UF Cardio-Vasculaire, Paris, France; 5 INRA, UMR1313 Génétique Animale et Biologie Intégrative, Jouy en Josas, France; 6 Centre for Sustainability, Environment and Health, National Institute for Public Health and the Environment, Bilthoven, Netherlands; 7 Inserm and Univ. Grenoble Alpes, U823, IAB Research Center, Team of Environmental Epidemiology Applied to Reproduction and Respiratory Health, Grenoble, France; 8 Institute of Risk Assessment Sciences, Utrecht University, Utrecht, Netherlands

## Abstract

**Background:**

Airborne pollution is a rising concern in urban areas. Epidemiological studies in humans and animal experiments using rodent models indicate that gestational exposure to airborne pollution, in particular diesel engine exhaust (DE), reduces birth weight, but effects depend on exposure duration, gestational window and nanoparticle (NP) concentration. Our aim was to evaluate the effects of gestational exposure to diluted DE on feto-placental development in a rabbit model.

Pregnant females were exposed to diluted (1 mg/m^3^), filtered DE (NP diameter ≈ 69 nm) or clean air (controls) for 2 h/day, 5 days/week by nose-only exposure (total exposure: 20 days in a 31-day gestation).

**Results:**

DE exposure induced early signs of growth retardation at mid gestation with decreased head length (*p* = 0.04) and umbilical pulse (*p* = 0.018). Near term, fetal head length (*p* = 0.029) and plasma insulin and IGF1 concentrations (*p* = 0.05 and *p* = 0.019) were reduced. Placental function was also affected, with reduced placental efficiency (fetal/placental weight) (*p* = 0.049), decreased placental blood flow (*p* = 0.009) and fetal vessel volume (*p* = 0.002). Non-aggregated and “fingerprint” NP were observed at various locations, in maternal blood space, in trophoblastic cells and in the fetal blood, demonstrating transplacental transfer. Adult female offspring were bred with control males. Although fetoplacental biometry was not affected near term, second generation fetal metabolism was modified by grand-dam exposure with decreased plasma cholesterol (*p* = 0.008) and increased triglyceride concentrations (*p* = 0.015).

**Conclusions:**

Repeated daily gestational exposure to DE at levels close to urban pollution can affect feto-placental development in the first and second generation.

**Electronic supplementary material:**

The online version of this article (doi:10.1186/s12989-016-0151-7) contains supplementary material, which is available to authorized users.

## Background

Diesel engine exhaust (DE) is composed of gases, including volatile and semi-volatile constituents, as well as particulate matter (PM), making up an important part of air pollution in European urban areas [1]. The impact of PM on mortality, cardiovascular and respiratory health in adulthood and also childhood is increasingly well characterized; more uncertainty exists regarding the effects of intra-uterine exposures [2, 3]. During pregnancy, maternal exposure to atmospheric pollution in humans may increase the risk of low birth weight concomitantly with an increase in particle concentration [4–6], with the smaller particular fraction corresponding to an aerodynamic diameter <2.5 μm (particulate matter 2.5 or PM_2.5_ being probably more harmful than the larger fraction (>2.5 μm)) [4]. Both increased and decreased placental weight and/or placental efficiency have been associated with PM exposure, depending on the geographical area [7, 8]. Studies in human populations, however, are limited in terms of the ability to explore toxicological mechanisms underlying the effects of DE on the developing fetus, and to explore the specific role of volatile fractions and associated PM.

To investigate this aspect, inhalation studies in animal models (mostly rodents) have been performed using various exposure times, various gestational windows and with varying concentrations of ultrafine particles, i.e. particles in the nanometer size range (NP), which are abundant in DE [9]. Observed biological effects of NP depend on their size, their composition and on the nature of the outer layer of proteins (corona) that forms during transport throughout the body [10]. The corona is considered important for both biocompatibility and transport in biological compartments [11] whereas the chemistry of NP also affects their biodistribution and biodegradation [12]. There is clear evidence from mice [13] and human ex vivo placental studies [14] that injected NP can cross the placental barrier and reach the fetus. In contrast, inhaled 11-15 nm cadmium oxide NP did not reach the fetus in a study in mice [15], suggesting that the inhalation route may not lead to the transfer of NP to the fetus.

Our aim was to observe the impact of maternal exposure to DE during gestation on fetoplacental development and transplacental transfer of NP. Our first hypothesis was that maternal DE exposure could have a negative impact on fetoplacental growth during gestation, and affect the fetal phenotype near term via effects on placental structure and vascularization. We also hypothesized that NP could cross the placenta to reach the fetal circulation. Rabbits were used as models because of their hemodichorial placentation, closer to the human placenta than that of rodents [16], their size which enables ultrasound monitoring during gestation and their short intergenerational period [17]. Nose-only exposure was used as it is more relevant to human exposure than the most often used whole body exposure, which does not discriminate effects due to inhalation from those due to ingestion after self-grooming.

## Methods

### Ethics

The local ethical committee (N°45 in the French National register) approved the experimentation under N°12/102.

### Animal exposure

**Fig. 1:**
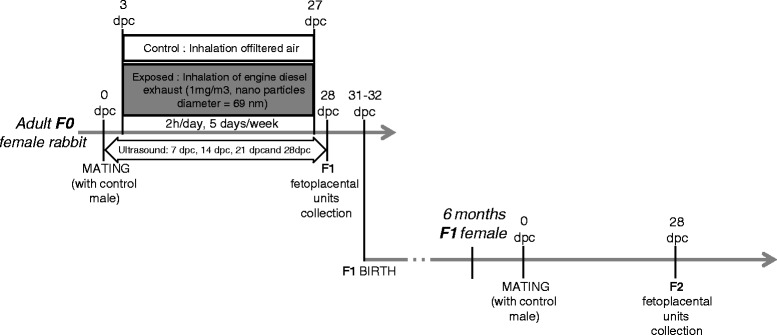
Experimental protocol over the 2 generations

Twenty-eight pregnant New-Zealand white female rabbits (INRA1077 line, 1-year old) (F0) were exposed by nose-only inhalation in custom made plexiglas tubes to either diluted DE (1 mg/m^3^) (exposed group) or clean air (control group) for 2 h/day, 5 days/week, from the 3rd to the 27th day post-conception (dpc) (i.e., 20 days altogether over a 31-day gestation) (Fig. 1). DE exposure was performed with the Mobile Ambient Particle Concentrator Exposure Laboratory [18] connected to a 25KVA Loxam engine, with a 500 nm particle filter (Additional file 1: Figure S1).

DE is a complex mixture of hundreds of constituents in either a gas or particle form. Gaseous components of DE include carbon dioxide, oxygen, nitrogen, water vapour, carbon monoxide, nitrogen compounds, sulphur compounds, and numerous low-molecular-weight hydrocarbons (some of them individually known to be toxic, such as aldehydes, benzene, 1,3-butadiene, polycyclic aromatic hydrocarbons (PAHs) and nitro-PAHS). The particles present in DE are known to be composed of center core of elemental carbon with absorbed organic compounds and small amounts of sulphate, nitrate, metals, and other trace elements [19]. The measured components of the exposure mixture in the present experiment are shown in Additional file 2: Table S1.

#### Ultrasound/Doppler monitoring

Twelve control and 16 exposed females were examined at 7, 14, 21 and 28 dpc using a Voluson E8 (GE Medical Systems) with a 6–18 MHtz linear probe (RSP6-16). B mode and 2/3D Doppler were performed transabdominally in 5 fetoplacental units/dam in non-sedated dams at 7, 14 and 21 dpc. On 28 dpc, 12 F0 dams were anaesthetized and a laparotomy was performed to analyze placental perfusion by quantitative tridimensional Power Doppler angiography (3D-PDA) [20], directly in contact with the pregnant horn [20, 21]. This procedure, enabling the precise measurement of whole placental perfusion, could only be performed just prior to sacrifice due to the need of a surgical procedure to access the pregnant horns. Perfusion was assessed through three indices: i) the Vascularization Index (VI), which represents the percentage of colored voxels representing the density of fetal vessels in the volume of interest; ii) the Flow Index (FI), that represents voxel intensity (from 1 to 100) depending on the intensity of blood flow and iii) the Vascularization Flow Index (VFI) that is a combination of VI and FI representing the blood perfusion. The relationship between these indices and real blood flow in the placenta was validated in sheep in our laboratory [21]. The high sensitivity of these indices was demonstrated in the pregnant rabbit using a pharmacological agent inducing placental vasoconstriction [20].

After euthanasia, maternal lungs, F1 (first generation) feto-placental units (41 control fetuses and 68 exposed fetuses), maternal and fetal plasma were collected. Functional zones of the placenta, i.e., the labyrinth (exchange area), the junctional zone and the decidua (maternal side) were dissected out. Fetuses and placentas were weighed; fetal length (crown to rump), fetal head (biparietal diameter and head length) and abdominal diameters were measured prior to dissection. Head measurements were performed using a digital caliper. Fetuses were sexed by visual observation of the internal genital organs.

Sixteen other females (*N* = 7 Controls, *N* = 9 Exposed) gave birth to F1 offspring, which were raised in control conditions. At 6.5 months of age, F1 females (*N* = 3 Controls, *N* = 9 in utero Exposed) were mated to control males and euthanized at 28 dpc to collect F2 (second generation) feto-placental units as described above.

#### Immunohistochemistry

Pieces of labyrinthine area were fixed with formalin. Samples were dehydrated in ethanol solutions, cleared in xylene, embedded in paraffin and then cut into 7 μm thick sections (Leica microtome, Germany). Immunodetection of vimentin was performed to label fetal capillaries on placental sections from the two groups (*N* = 10 Control and *N* = 10 Exposed placentas) as described previously [22].

#### Stereological analysis

After immunodetection, all placental sections were scanned using a NanoZoomer Digital Pathology System (NDP Scan U100074-01, Hamamatsu, Japan). Volume fraction and surface density of the components of the labyrinthine area, i.e. trophoblast, fetal vessels and maternal blood space were quantified using the One Stop Stereology method available on the Mercator® software, as described previously [23].

#### TEM analyses

Labyrinthine area and lung samples collected randomly from 4 fetuses in each litter were fixed with 2 % of glutaraldehyde overnight at 4 °C and then washed 3 times with sodium cacodylate-buffer. Samples were post-fixed in 0.5 % osmium tetroxide, dehydrated using a series of ethanol dilutions and embedded in Epon resin. Sections (0.5 μm) were stained with Toluidine Blue and examined with an Olympus microscope. Ultrathin sections (75 nm) were stained with lead citrate and examined with a Zeiss EM902 EELS transmission electron microscope.

#### Biochemistry

Classical biochemistry (triglycerides, cholesterol, glycemia, urea, creatinin, ASAT, ALAT, insulin) was performed on plasma using Beckman Coulter equipment. IGF1 was analyzed by ELISA [24].

#### Statistical analysis

Data are expressed as: median [Q1; Q3], with first (Q1) and third quartile (Q3) corresponding to 25 and 75 % of scores, respectively. For all data but ultrasound analyses at 7, 14 and 21 days, a linear model was used, with random effect of dam adjusted for treatment, litter size, fetal position in the horn (indexed in 3 categories) and fetal sex using the linear mixed effects model (nlme package, R, Pinheiro, Bates, DebRoy, Sarkar and the R Development Core Team 2013. nlme. R package version 3.1-111; www.r-project.org/). For ultrasound analyses at 7, 14 and 21 days, the linear model was adjusted only for the dam as the other parameters (sex, position in the horn) were not available.

## Results

### Intra-uterine growth of the F1 generation with Ultrasound/Doppler monitoring

No sex-specific difference was observed in any of the analyses.

At 7 dpc, no effect of exposure to DE was observed on embryo diameter, perimeter and volume (Additional file 3: Table S2).

At mid-gestation (14 dpc), head length (−9.6 %, *p* = 0.04) and umbilical pulse (−3.6 %, *p* = 0.018) were significantly reduced in exposed fetuses vs. controls (Table 1). Other fetal developmental parameters (crown-rump length, body and head width, body perimeter) were not affected.

**Table 1:** Ultrasound measurements at 14 dpc

Variable	Number of fetuses		Median [Q1; Q3]		Adjusted p-value		
	C	E	C	E	β value	CI	*P*-value
Crown-rump length (mm)	35	65	11.3 [10.8; 11.8]	11.2 [10.8; 11.8]	−0.275	[−0.821;0.271]	0.332
Body width (mm)	35	64	5.0 [4.7; 5.2]	5.0 [4.7; 5.2]	0.009	[−0.245;0.263]	0.945
Head length (mm)	36	64	8.4 [7.5; 9.15]	8.2 [6.9; 8.6]	−0.59	[−1.116;-0.064]	0.040*
Head width (mm)	36	64	4.3 [4.0; 4.6]	4.2 [3.9; 4.5]	−0.107	[−0.29;0.076]	0.261
Body perimeter (mm)	35	65	68 [63.9; 70.3]	67.4 [64.9; 69.8]	0.902	[−2.457;4.26]	0.602
Umbilical pulse (beat/min)	33	65	222 [219.5; 227.5]	214.5 [208.8; 218.6]	−8.807	[−15.527;-2.088]	0.018*

The effects of gestational DE exposure on fetal body and head parameter (length, width and perimeter) and on umbilical pulse were measured with Doppler ultrasound in control (C) and exposed (E) groups. The linear model was adjusted only for the dam and all data are expressed as median [Q1; Q3] (*p < 0.05, compared with control group)

At 21 dpc, DE exposure was not associated any more with any variation in abdominal perimeter, femur and head length, biparietal diameter, heart rate, nor Doppler (umbilical resistance index and systolic and diastolic velocities) or placental measurements (Additional file 4: Table S3 and Additional file 5: Table S4).

At 28 dpc, i.e., 3 days before birth, umbilical cord and fetal cerebral artery Doppler parameters were not affected by maternal exposure (Additional file 6: Table S5). Head length, however, was significantly decreased (−4 %, *p* = 0.029) and abdominal perimeter tended to be decreased (−4 %, *p* = 0.076) in exposed fetuses compared to controls. Placental efficiency, which is defined as the fetal to placental weight ratio, i.e., the number of grams of fetus per gram of placenta, was significantly decreased (−12.3 %, *p* = 0.049) in the exposed group compared to controls. Other biometric parameters (fetal weight, crown-rump length, biparietal diameter) as well as organ (brain, lung, heart, liver and kidney) to fetal weight ratios and placental parameters (total placental weight, weight of the labyrinthine area and of the decidua) did not vary significantly with DE exposure (Table 2).

**Table 2:** Fetoplacental biometry at 28 dpc for the first generation

Variable	Number of fetuses		Median [Q1; Q3]		Fully adjusted linear model		
	C	E	C	E	β value	CI	*P*-value
Fetal weight (g)	41	68	38.5 [36.0; 41.7]	34.45 [31.1; 37.8]	−1.895	[−5.362;1.571]	0.307
Crown-rump length (cm)	41	68	12.0 [11.6; 12.5]	11.7 [11.1; 12.1]	−0.107	[−0.494;0.28]	0.596
Biparietal diameter (mm)	41	66	17.9 [17.5; 18.2]	17.2 [16.3; 17.8]	−0.697	[−1.649;0.256]	0.178
Head length (mm)	41	66	29.5 [28.7; 30.2]	28.4 [27.4; 29]	−1.293	[−2.301;-0.2285]	0.029*
Abdominal perimeter (cm)	41	67	7.5 [7.3; 8.0]	7.2 [6.8; 7.5]	−0.529	[−1.058;0]	0.076
Brain weight (g)	40	68	0.92 [0.88; 0.96]	0.93 [0.86; 0.96]	0.011	[−0.055;0.077]	0.746
Brain/Fetus weight ratio	40	68	0.025 [0.023; 0.028]	0.027 [0.025; 0.030]	0.002	[−0.001;0.004]	0.241
Lung/Fetus weight ratio	39	66	0.029 [0.027; 0.031]	0.030 [0.028; 0.032]	0	[−0.001;0.002]	0.570
Heart/Fetus weight ratio	41	68	0.006 [0.005; 0.006]	0.005 [0.005; 0.006]	0	[0;0.001]	0.662
Liver/Fetus weight ratio	41	68	0.066 [0.063; 0.072]	0.068 [0.062; 0.071]	−0.002	[−0.007;0.003]	0.514
Kidney/Fetus weight ratio	39	64	0.0006 [0.0006; 0.0007]	0.0006 [0.0005; 0.0007]	0	[0;0]	0.999
Placental weight (g)	41	68	7.32 [6.64; 8.43]	7.51 [6.83; 8.57]	0.153	[−0.794;1.1]	0.756
Labyrinthine area weight (g)	41	66	5.03 [4.41; 5.92]	4.99 [4.34; 6.05]	−0.17	[−1.003;0.663]	0.695
Decidual weight (g)	41	66	2.16 [1.98; 2.56]	2.51 [2.14; 2.96]	0.307	[−0.164;0.778]	0.226
Placental efficiency	41	68	5.29 [4.65; 5.51]	4.64 [3.92; 5.00]	−0.63	[−1.187;-0.074]	0.049*

Female rabbits inhaled 1 mg/m^3^ of NP, 2 h/day, 5 days/week, from 3 dpc to 27 dpc. Dams were euthanized and fetoplacental units of control (C) and exposed (E) group were collected at 28 dpc. Effect of pregnancy exposure to engine diesel exhaust on first-generation fetuses was estimated using a linear model with random effect of dam adjusted for litter size and position of the fetus in the horn. All data are expressed as median [Q1; Q3] (*p < 0.05, compared with control group)

Fetal plasma insulin (−44.4 %, *p* = 0.05) and IGF-1 (−91 %, *p* = 0.019) concentrations were significantly reduced in exposed compared to control fetuses at 28 dpc (Table 3).

**Table 3:** Fetal metabolism at 28 dpc for the first generation

Variable	Number of fetuses		Median [Q1; Q3]		Fully adjusted linear model		
	C	E	C	E	β value	CI	*P*-value
Glycemia (mmol/L)	16	20	4.250 [3.200; 4.675]	4.210 [3.150; 5.358]	0.401	[−0.794;1.597]	0.521
Insulin (mUI/L)	17	19	1.800 [1.300; 2.950]	1.000 [0.700; 1.400]	−0.998	[−1.886;-0.111]	0.050*
Total cholesterol (mmol/L)	17	20	1.950 [1.640; 2.620]	2.115 [1.818; 2.455]	0.013	[−0.436;0.462]	0.956
HDL cholesterol (mmol/L)	17	20	0.460 [0.433; 0.498]	0.400 [0.360; 0.490]	0.035	[−0.03;0.099]	0.309
Non-HDL cholesterol (mmol/L)	17	20	1.590 [1.275; 2.165]	1.645 [1.388; 1.948]	−0.022	[−0.412;0.369]	0.915
Triglycerids (mmol/L)	15	20	0.570 [0.460; 0.670]	0.585 [0.445; 0.870]	0.062	[−0.238;0.362]	0.689
ASAT (UI/L)	17	20	168.0 [86.50; 184.5]	171.5 [129.3; 217.5]	26.413	[−19.769;72.594]	0.284
ALAT (UI/L)	10	16	7.000 [6.000; 9.500]	9.000 [7.000; 9.750]	1.18	[−1.068;3.428]	0.326
Creatinine (μmol/L)	17	20	87.00 [84.50; 91.00]	95.00 [92.00; 104.8]	7.329	[−3.003;17.662]	0.190
Urea (mmol/L)	17	20	5.600 [5.150; 5.850]	6.500 [5.425; 7.450]	0.841	[−0.369;2.05]	0.199
IGF-1 (nmol/L)	20	20	1.184 [0.337; 2.368]	0.108 [0.000; 1.581]	−0.105	[−0.179;-0.031]	0.019*

Effect of exposure to engine diesel exhaust on first-generation fetuses was estimated using a linear model with random effect of dam adjusted for litter size and position of the fetus in the horn. All data are expressed as median [Q1; Q3] (*p < 0.05, **p < 0.01, compared with control group)

*ASAT* ASpartate Amino Transferase, *ALAT* ALanine Amino Transferase

Contemporary to the groups used for fetoplacental exploration, two groups of exposed (*N* = 7) and control (*N* = 9) dams (F0) were allowed to give birth. At birth, litter weight tended to be lower in exposed vs. control dams (−18.3 %, *p* = 0.065) (Additional file 7: Table S6).

### Materno-placental exchanges and placental vascularization

#### Quantitative Power Doppler analysis of placental vascularization at 28 dpc

**Fig. 2:**
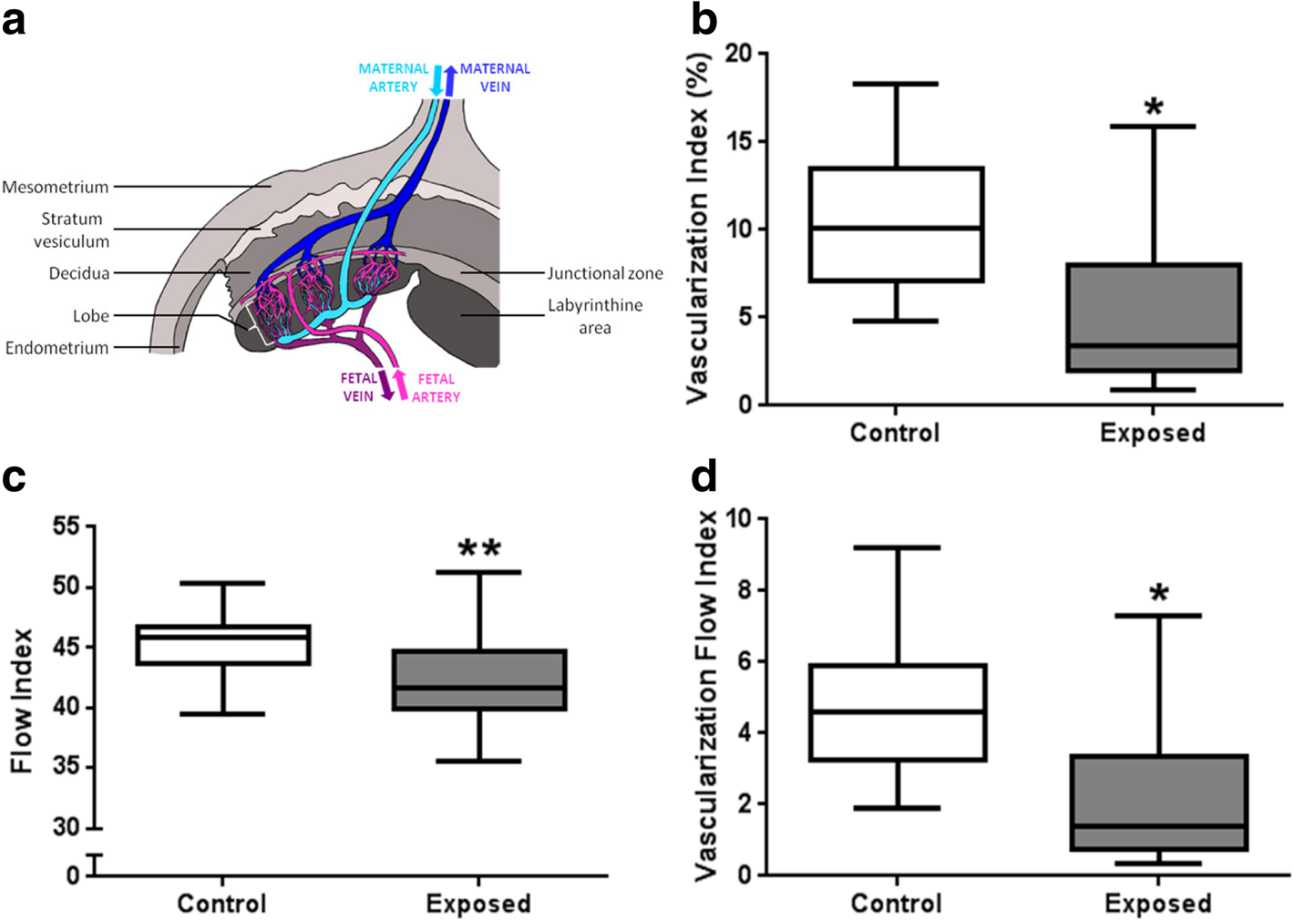
Ultrasound monitoring of placenta at 28 dpc. Drawing showing the rabbit placental structure and blood flow (**a**). 3D power Doppler was performed on dams to determine the Vascularization Index (**b**), the Flow Index (**c**) and the Vascularization Flow Index (**d**) at 28 dpc. All data are expressed as median [Q1; Q3] (*p < 0.05, **p < 0.01, compared with control group)

Near term, DE exposure induced reductions in VI (−66.1 %, *p* = 0.017), FI (−9.3 %, *p* = 0.009) and VFI (−70.1 %, *p* = 0.015) (Fig. 2b, c, d). Altogether these data show that maternal exposure to diluted filtered DE induced a reduction in placental blood flow near the term.

#### Placental stereology

**Fig. 3:**
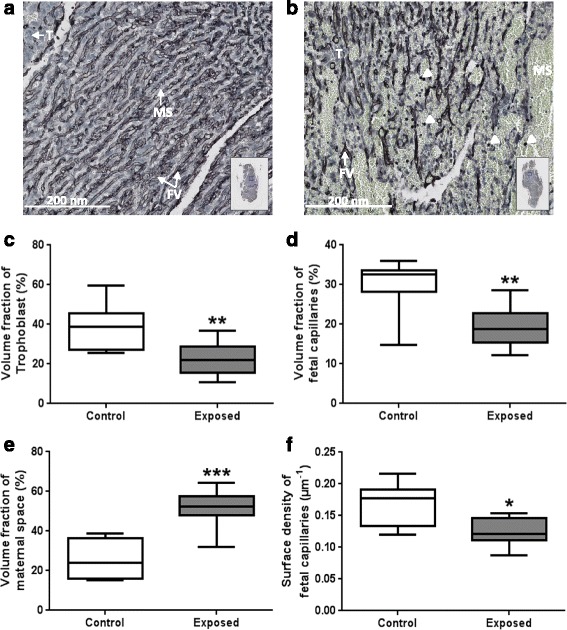
Stereological examination of placenta at 28 dpc. At 28 dpc, immunodetection of vimentin to label fetal capillaries was performed on labyrinthine area sections from the control group (**a**) and the exposed group (**b**). Black immunostaining represents fetal vessels (FV), blue cells are trophoblasts cells (T) and the white head arrows show circulating cells in maternal space (MS). Volume fraction of trophoblast (**c**), fetal capillaries (**d**) and maternal blood space (**e**) and surface density of fetal capillaries (**f**) were quantified. All data are expressed as median [Q1; Q3] (*p < 0.05, **p < 0.01, ***p < 0.001, compared with control group)

The rabbit placenta develops as a labyrinthine and hemodichorial placenta close to that of the human (villous and hemochorial) [16] and is made up of three compartments: the decidua (of maternal origin), and the junctional and labyrinthine areas of fetal origin (Fig. 2a). Materno-fetal exchanges take place in the labyrinthine area, which cellular composition was explored by stereology (Fig. 3a and b). At 28 dpc, the relative volume fractions of trophoblastic cells (−43 %, *p* = 0.004) and fetal capillaries (−42 %, *p* = 0.002) were significantly decreased in exposed placentas compared to controls (Fig. 3c and d). The relative surface density of fetal capillaries was significantly reduced in the labyrinthine area (−31.7 %, *p* = 0.014) from exposed compared to control fetuses (Fig. 3f). In contrast, the relative volume of maternal blood space was significantly larger (+116 %, *p* = 0.0001) in the exposed group compared to controls (Fig. 3e). The relative volume (+370 %, *p* = 0.001) and surface of circulating immune cells (+469 %, *p* = 0.001) in the maternal space were significantly increased in exposed placentas compared to controls (Additional file 8: Table S7).

These measurements confirmed the in vivo observations made with 3D-PDA.

### Transplacental transfer of NP

To determine the ability of inhaled diesel NP to reach the fetus, ultrathin sections of maternal lungs and placental labyrinthine area collected at 28 dpc were examined by Transmission Electron Microscopy (TEM). Since the identification of NPs in the placental tissues was only based on TEM observations, the observed black particles are described below as “NP-like”.

NP-like were observed in lungs of exposed does, located specifically in type 1 pneumocytes within both the cytoplasm and the nucleus (Additional file 9: Figure S2). Isolated, non-aggregated NP-like were also observed within blood vessels (in maternal erythrocytes and plasma) (Additional file 10: Figure S3).

**Fig. 4:**
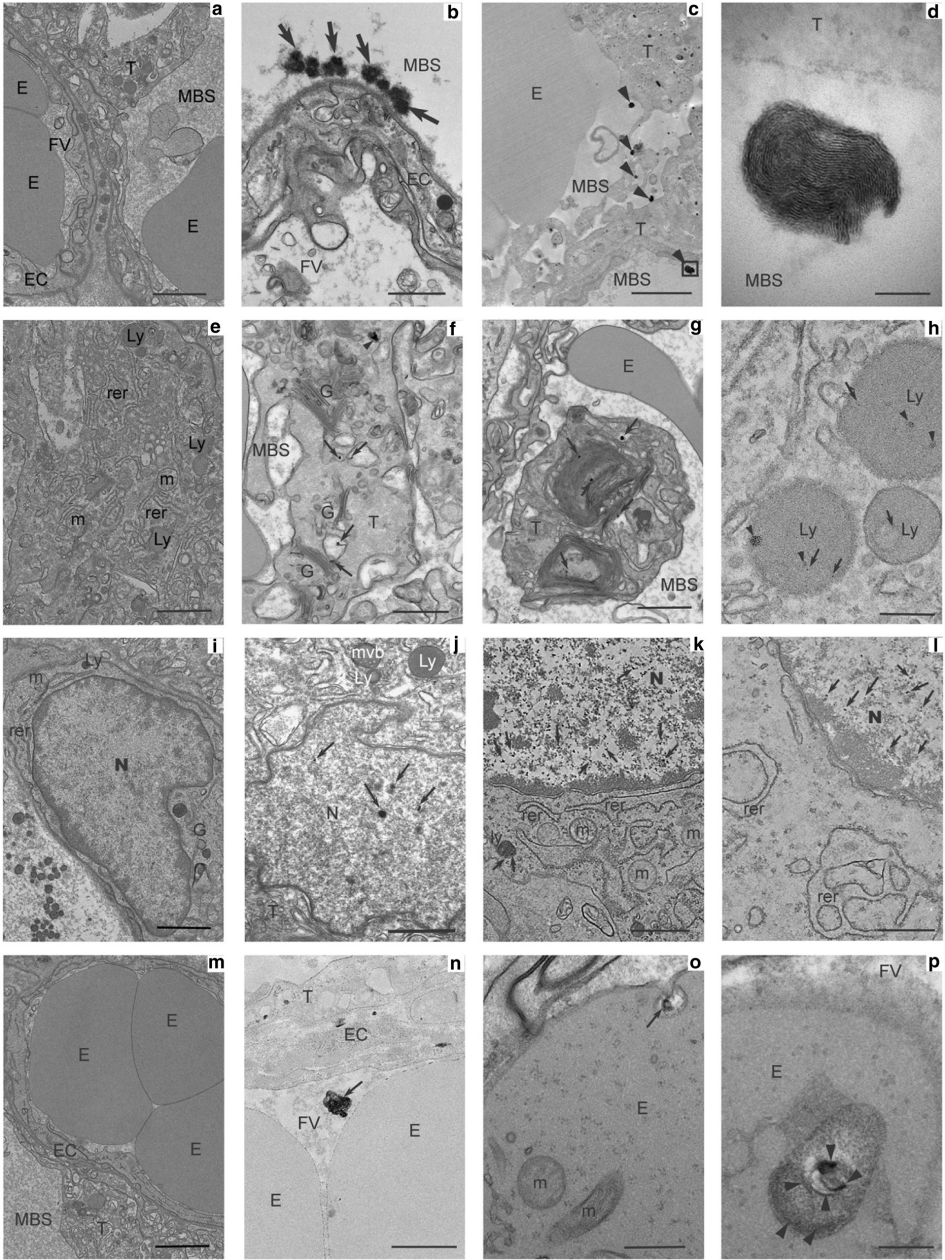
Localization of nanoparticles in the placenta at 28 dpc. Ultrathin sections were performed on labyrinthine area in placentas from control (**a**, **e**, **i**, **m**) and exposed (**b**-**d**, **f**-**h**, **j**-**l**, **n**-**p**) dams and analyzed by TEM. Arrows indicate nanoparticles and arrowheads “finger-print” like particles. Several observations were made with various magnifications allowing the observations of different cellular compartments. Scale bars: **a**: 2 μm; **b**: 800 nm; **c**: 2 μm; **d**: 100 nm; **e**: 1.6 μm; **f**: 700 nm; **g**: 1.25 μm; **h**: 1 μm; **i**: 1.6 μm; **j**: 1 μm; **k**: 1 μm; **l**: 700 nm; **m**: 2.5 μm; **n**: 1 μm; **o**: 400 nm; **p**: 20 nm. Abbreviations: E: Erythrocyte; EC: Endothelial Cell; FV: Fetal Vessel; G: Golgi apparatus; Ly: Lysosome; m: mitochondria; MBS: Maternal Blood Space; mvb: multivesicular body; N: Nucleus; rer: rough endoplasmic reticulum; T: Trophoblast

In the placenta, diffuse aggregated NP-like were observed along the microvilli of syncytial membranes by TEM analysis in the maternal blood spaces of exposed placentas (Fig. 4b), while no NP-like could be identified in control placentas (Fig. 4a). Free “finger-print” like NP-like were also found in maternal blood spaces (Fig. 4c and d). In the cytoplasm of trophoblastic cells, isolated NP-like were located in endosomes (Fig. 4f), in figures of autophagy (Fig. 4g) and in lysosomes (Fig. 4h). No similar observation was made in control placentas (Fig. 4e). Compact NP-like were observed scarcely scattered in the nucleus of some trophoblastic cells whose cytoplasm was well-preserved (Fig. 4j). In contrast, numerous small NP-like (diameter 40 ± 10 nm, *N* = 60) were present in the nuclei of other trophoblastic cells (Fig. 4k and l), together with morphological organelle abnormalities indicating cellular death (Fig. 4k and l). This was not observed in control animals (Fig. 4i). Other “finger-print” like particles were also observed in the trophoblast (data not shown). Aggregated NP-like were visualized in the lumen of fetal vessels from exposed animals (Fig. 4n) but not in controls (Fig. 4m). Finally, NP-like were observed in endocytotic vesicles in fetal erythrocytes (Fig. 4o) with some suggestion of on-going formation of “finger-print” like structures (Fig. 4p). The presence of numerous organelles in the fetal erythrocytes from exposed dams suggests a delay in erythrocyte maturation as erythrocytes should contain only hemoglobin at 28 dpc [25].

### Fetoplacental biometry and metabolism of second generation (F2)

At adulthood, F1 female rabbits (6.5 months) were mated to generate a second generation. F2 fetoplacental units were collected at 28 dpc.

F0 exposure had no effect on F2 fetal biometry (fetal weight, crown-rump and head length, biparietal diameter and abdominal perimeter). Organs-to-fetal weight ratios were similar in the two groups for all considered organs (brain, lungs, heart, liver and kidneys). There was no significant effect on placental biometry (placental weight, labyrinthine area weight, decidual weight and placental efficiency) (Additional file 11: Table S8). Conversely, in “exposed” F2 fetuses, plasma triglyceride concentrations were significantly increased (+25.9 %, *p* = 0.015) whereas total fetal plasma cholesterol and non-HDL cholesterol concentrations were significantly decreased (−25.9 %, *p* = 0.008 and −26 %, *p* = 0.007, respectively) compared to controls (Table 4).

**Table 4:** Fetal metabolism at 28 dpc for the second generation

Variable	Number of fetuses		Median [Q1; Q3]		Fully adjusted linear model		
	C	E	C	E	β value	CI	*P*-value
Glycemia (mmol/L)	14	12	1.400 [1.175; 1.765]	1.730 [1.618; 2.070]	0.257	[−0.173;0.687]	0.275
Insulin (mUI/L)	14	12	4.170 [1.990; 10.09]	2.545 [1.703; 3.765]	−2.965	[−6.245;0.315]	0.111
Total cholesterol (mmol/L)	15	12	2.550 [2.180; 2.640]	1.890 [1.800; 2.240]	−0.416	[−0.648;-0.183]	0.008**
HDL cholesterol (mmol/L)	15	12	0.730 [0.685; 0.790]	0.650 [0.590; 0.750]	−0.071	[−0.156;0.015]	0.143
Non-HDL cholesterol (mmol/L)	15	12	1.730 [1.480; 1.898]	1.280 [1.150; 1.480]	−0.348	[−0.54;-0.156]	0.007**
Triglycerids (mmol/L)	15	12	0.540 [0.440; 0.598]	0.680 [0.610; 0.730]	0.176	[0.062;0.289]	0.015*
ASAT (UI/L)	15	10	107.5 [97.00; 116.0]	110.0 [95.00; 137.0]	15.66	[−14.542;45.863]	0.335
Creatinine (μmol/L)	15	12	72.00 [66.00; 76.25]	72.00 [67.00; 77.00]	0.082	[−6.745;6.909]	0.982
Urea (mmol/L)	15	12	4.650 [3.950; 5.200]	3.700 [3.500; 4.600]	−0.303	[−1.779;1.174]	0.696

The effects of exposure to engine diesel exhaust during grand-mother pregnancy on second-generation fetuses were estimated using a linear model with random effect of dam adjusted for litter size, fetal sex and the position of the fetus in the horn. All data are expressed as median [Q1;Q3] (*p < 0.05, **p < 0.01, compared with control group)

## Discussion

### Exposure to DE

The DE exposure (1 mg/m^3^, 2 h/day, 5 days/week for 20 days over 31-day gestation, with mean diameter = 69 nm) in the present study equates to a mean exposure of 80 μg/m^3^ over 24 h, which is much higher than the 25 μg/m^3^ WHO daily average recommendation for PM_2.5_ [26], whereas there are no recommendations for NP. This DE exposure is similar to the daily human exposure in large European areas when people drive or walk on big roads twice a day. It could be possible that a daily 2 h exposure at 1 µ gram/m^3^ may have a different impact than a same pulmonary dose delivered over a 24 h period, but most studies consider the accumulation effect. Several studies in rats and mice, however, have been published in which similar or even higher doses and concentrations were used for longer exposure durations without observing moderate to severe detrimental effects in the lung [27, 28].

### Fetal growth

In this study, DE exposure induced signs of growth retardation at mid-pregnancy in the first generation, with a reduction in head length and umbilical pulse, suggesting fetal distress, which is in agreement with previous observations in humans [5]. In the present rabbit model, although signs of hypotrophy were observed by ultrasound at 14 dpc, no hypotrophy was observed at 21 dpc, suggesting that catch-up growth occurred in the second half of pregnancy. This is possibly due to placental adaptations aimed at overcoming growth retardation as described in the context of maternal over nutrition in mice, where initial IUGR induced by maternal excess nutrition around mid-pregnancy was subsequently compensated for by an increase in the expression of placental transporters [29]. Here, on 28 dpc, i.e., 3 days before term, however, the decreased placental blood flow due to reduced placental vascularization was associated with a limited hypotrophy, i.e., a reduction in head length with a tendency for a reduced abdominal perimeter, which indicates that these adaptations were not sufficient to sustain fetal growth up to term. Overall, these results are in agreement with those of Slama et al. [5], in humans, showing decreased biparietal diameter in relation to maternal individual exposure to benzene (a marker of traffic-related pollutants in non-smoking women) in the second and third trimester of pregnancy, measured by ultrasound. Effects on head circumference at birth have also been observed for PM_2.5_ in other human studies [4].

The reduced fetal head length associated to a weak decrease of the abdominal perimeter at 28 dpc may, as well as the reduced placental efficiency, suggest that placental compensation mechanisms that developed at mid-gestation became insufficient to maintain optimal fetal development near term, as observed in protein-restricted pregnant mice [30].

Fetal insulin is known to act as a growth factor [31]. Hypoinsulinemia correlated with a decrease in IGF-1 concentration, as observed here in F1 fetuses, has been associated with growth retardation in rat fetuses [31]. These observations are also supported by another study in rabbits showing a positive correlation between plasma insulin concentrations and fetal bodyweight, reflecting the involvement of insulin in the regulation of fetal growth [32]. In IUGR humans, hypoinsulinemia was only observed at adulthood [33].

In animal models, controversial effects of DE inhalation on feto-placental development have been reported (See exposure details in Table 5). In rats, mice and rabbits, the United States Environment Protection Agency tested the effects of concentrations of 6 to 12 fold that of limit values for DE exposure in humans during the first half of gestation and found no effects on fetal survival and development [9]. In contrast, other studies have shown that high gestational exposure throughout pregnancy [34] or exposure to moderate concentrations during the first week of gestation [35] can decrease fetal [34, 35] and sometimes also placental weights [35]. Paradoxically, in rats, an increase in fetal weight in females only and a reduction in placental weight in males only were observed after exposure to very high concentrations of DE [36]. Such an increase in fetal weight, together with a decrease in crown-rump length was also reported in rats exposed to low concentrations of DE [37]. These different observations might be explained by differences in animal models (species, strain), gestational window of exposure, timing and duration of daily exposure, particle size (filtered or total DE) or particle concentration. No clear mechanistic explanation, however, can be inferred from these studies.

**Table 5:** Animal studies with DE exposure

Animals	Exposure		References
	Concentration	Duration	
SD rats	10 % DE 6 mg/m^3^ NP	6-15 dpc 8 h/d, 7d/w	(9)
New Zealand rabbits	10 % DE 6 mg/m^3^ NP	6-18 dpc 8 h/d, 7d/w	(9)
CD-1 mice	DE 12 mg/m^3^ NP	over 3 generations	(9)
Slc: ICR mice	DE 3 mg/m^3^ NP	2-13 dpc 12 h/d, 7d/w	(34)
Swiss mice	DE 42 μg/m^3^ NP	all gestation 24 h/d, 7d/w	(35)
F344 rats	Total DE 5.63 mg/m^3^ NP	7-20 dpc 6 h/d, 7d/w	(36)
Fisher rats	DE 148.86 μg/m^3^ NP	1-19 dpc 5 h/d, 7d/w	(37)
C57 Bl/6 J mice	DE 300 μg/m^3^ NP	0.5-17 dpc 6 h/d, 7d/w	(38)
BalbC mice	DE 27.5 μg/m^3^ NP	over 3 generations	(48)
BalbC mice	DE 27.5 μg/m^3^ NP	over 3 generations	(47)
New Zealand rabbits	DE 1 mg/m^3^ NP	3-27 dpc 2 h/d, 5d/w	Present study

All these studies were cited in Discussion

### Placental structure and vascularization

Fetal growth is controlled by placental function, and particularly dependent on placental perfusion. In this study, placental vascularization was decreased with a reduction in relative volume and surface of fetal vessels. This reduction in placental vascularization and perfusion as well as the presence of NP-like in all placental compartments, and in particular on the microvillous membrane, could contribute to the reduced nutrient exchanges between the maternal and the fetal blood. Moreover, using TEM, we observed trophoblastic cells with nuclei filled with NP-like together with altered organelle structure (data not shown), suggesting cell degeneration. Although they could not be quantified (in terms of number of cells affected), placental function could possibly be affected. In mice, in utero exposure to 300 μg/m^3^ (6 h/day, 5 days/week) of inhaled DE did not affect fetal weight but decreased placental weight [38]. The authors mentioned an increase in stromal density (not quantified) which could have induced a reduction in the labyrinthine vascular space, in agreement with our observations [38]. In the present study, placental perfusion, as measured by 3D-PDA, was affected by DE maternal exposure and associated with the decreased vascular density observed through the stereological approach [20]. IUGR and decreased placental vascularization were also observed in a mice study with maternal exposure to PAH before conception [39]. In terms of effects of DE on placental function, the placental immune function has been explored [40] but so far, in vivo or histological exploration of placental vascularization as performed in the current study have, to our knowledge, rarely been performed. Moreover, most studies used rodent models, for which the placenta is not as close to that of humans compared to rabbits [16, 17, 41, 42].

### Transplacental transfer of NP

The transplacental transfer of NP has been demonstrated using animal models mostly after intravenous or intraperitoneal NP injections [11, 13, 43]. Blum et al. [15], however, showed that inhaled cadmium oxide NP could reach the placenta but not the fetus. In the present study using inhaled NP-rich DE, NP-like were observed in lungs of exposed does in type 1 pneumocytes and within blood vessels (in maternal erythrocytes and plasma), which we assume to be of DE origin. This would demonstrate that NP are able to cross the lung barrier and reach maternal organs, including the placenta. Indeed, in the cytoplasm of placental trophoblastic cells, similar NP-like were observed as isolated structures in endosomes, in figures of autophagy and in lysosomes, indicating that endocytosis may be one way for the transplacental transfer of NP. Moreover, we demonstrated that NP-like are able to reach the fetal erythrocytes, as also shown by Soler et al. [44]. Nevertheless, the mechanism through which NP-like cross the placenta remains unknown and may depend on their chemical composition [43]. We hypothesize that NP-like could cross placenta by endocytosis, but could also be conveyed through by simple diffusion or facilitated transport, also depending on their size. This deserves to be further investigated. Moreover, the different forms for isolated, aggregated NP-like or “finger-print” like particles observed in the placenta could reflect an ongoing transformation process of the inhaled NP, in relation to the repeated exposure throughout gestation. Small NP-like could be the result of recent DE exposure whereas the “finger-print” like particles could result from the degradation of NP inhaled at the beginning of the experiment. These “finger-print” like particles have been previously observed in mouse spleen and liver after injection of magnetic iron NP and have been suggested to result from a lysosomal degradation process [45]. Further analyses will be performed to determine the chemical composition of these NP in order to confirm that they originate from inhaled diesel exhaust and study if iron metabolism is involved in this degradation.

### Intergenerational effects

At birth, exposed F1 litter weight tended to be lower compared to controls. They had caught up at adulthood (data not shown). Whether intergenerational effects could be conveyed through contamination of the maternal milk remains to be determined. Nevertheless, so far, preliminary studies on the maternal mammary gland do not provide clear evidence of the presence of NP in mammary tissue (data not shown).

In this study, intergenerational effects were observed on the second generation. The plasma concentrations of triglycerides were higher and that of cholesterol were lower in “exposed” F2 fetuses compared to controls, indicating metabolic dysfunction. This metabolic impairment during* in utero* life could predispose offspring to the onset of metabolic syndrome at adulthood, as shown by Picone et al. [46] and Tarrade et al. [47] in a context of maternal high fat diet in rabbits. Moreover, in a study in mice, cumulative effects were observed after continuous exposure of males and females to Sao Paulo air pollution for 3 generations: in the third generation, IUGR and a reduction in volume, caliber and surface of maternal placental space together with an increase in placental fetal vessel surface were observed [48, 49]. These experiments, however, did not discriminate between pre-conceptional, gestational and postnatal effects, nor between paternal and maternal effects. In the present study, the intergenerational effects, as observed in F2 fetuses, could be due to metabolic modifications during fetal and post-natal growth of the F1 animals and thus could possibly not be directly caused by NP but result from indirect effects.

### Relative role of NP and other DE components

Altogether, the effects observed here could be due to either NP or Polycyclic Aromatic Hydrocarbons (PAH) among other volatile components and most probably to the combined effects of several components of the mixture composing DE. Indeed, one study in humans compared the vascular effects of the inhalation of total DE, filtered DE or pure carbon nanoparticulate in healthy adult patients [50]. Only total DE significantly affected plasma concentrations of vasodilators, whereas filtered DE and pure carbon nanoparticulate did not have a significant effect [50]. The effect of the combined elements could be due to the adsorption of the volatile compounds on the corona of the NP.

### Strengths and limitations

Rabbits were used because of their hemodichorial placenta closer to humans than those of rodents, with a body size enabling ultrasound monitoring during gestation like in humans. Moreover, genetic diversity is maintained in rabbits as opposed to the inbred genetic lines available in rodents. This experiment focused on feto-placental development of the first and the second generations after exposure of F0 females but did not aim at studying intergenerational effects (involving the F3). The technical option of nose-only exposure was selected in order to avoid oral absorption of NP through self-grooming. The use of filters blocking particles with a diameter larger than 500 nm mimicked the diesel particulate filters installed in current diesel vehicles.

Nevertheless, the results obtained here should be confirmed in other studies in humans and/or animals. Rabbits, like rodents, are polycotous animals, in contrast to the unique or twin pregnancy in humans. Their gestation length is short (31 days), thus reducing the likelihood to observe chronic adaptive mechanisms as could be developed during a 9 month long human pregnancy. Furthermore, dams were exposed twice a day to a peak concentration rather than being exposed to lower concentrations throughout the day. Our experimental model is also not totally applicable to the human situation because exposure occurred only during gestation with no preconceptional or postnatal exposure. The sample size was also obviously limited by technical constraints. Our choice was not to formally rely on significance testing, but to look for coherent patterns in associations with DE exhaust. Finally, we have not so far characterized the chemical composition of NP, even though a carbon core is most probable according to the literature. We are aware that, given the novelty of the field, our study represents a first step to highlight potential risk factors for birth outcomes, and should be seen as hypothesis-generating. These hypothesis need to be confirmed by future studies.

## Conclusion

The data presented in this paper demonstrates that placental function is disturbed by maternal exposure to DE rich in NP, especially through a reduced placental vascularization in rabbits. Fetal growth is affected, in agreement with the limited observational studies in humans [8]. Decreased fetal plasma insulin and IFG1 concentrations are in agreement with data from other animal models [31]. Moreover, NP from DE are able to cross the placenta and reach the fetal circulation, although limited effects were observed in the fetus in the present study. Nevertheless, the toxicity of inhaled DE on offspring health should be further explored up to adulthood. DE exposure affects fetal metabolism in the second generation, thereby demonstrating intergenerational effects.

Altogether, these data indicate that during pollution peaks, pregnant women, and not only infants and elderly people, should be considered as a high risk population. Atmospheric pollution should be taken into account, as well as maternal nutrition, bodyweight, stress, pharmaceutical treatment, as a disruptor on offspring’s phenotype establishment at adulthood.

## Abbreviations

3D-PDA,Tridimensional Power Doppler Angiography; DE, diesel engine exhaust; dpc, days post-conception; F1, first generation; F2, second generation; FI, Flow Index; IGF1, Insulin-like Growth Factor 1; IUGR, Intra-Uterine Growth Restriction, NP, nanoparticles; PAHs, Polycyclic Aromatic Hydrocarbons; PM, Particulate Matter; TEM, Transmission Electron Microscopy; VI, Vascularization Index; VFI, Vascularization Flow Index; WHO, World Health Organization.

## Additional files

Additional file 1: Figure S1. Particle size distribution during exposure. (PPTX 52 kb)

Additional file 2: Table S1. Diesel exhaust composition during exposure. NO: Nitrogen Oxid, CO: Carbon Oxid. (PPTX 46 kb)

Additional file 3: Table S2. Ultrasound embryo measurements at 7 dpc in first generation. Ellipse was used to measure embryo diameters. Diameter 1 represents the longest one and Diameter 2 the shortest one. All data are expressed as median [Q1;Q3]. (PPTX 51 kb)

Additional file 4: Table S3. Ultrasound fetal measurements at 21 dpc in the first generation. All data are expressed as median [Q1;Q3]. (PPTX 63 kb)

Additional file 5: Table S4. Ultrasound placental measurements at 21 dpc. Mean Grey represents the density of tissu of interest. All data are expressed as median [Q1;Q3]. (PPTX 46 kb)

Additional file 6: Table S5. Ultrasound fetal measurements at 28 dpc. All data are expressed as median [Q1;Q3]. (PPTX 64 kb)

Additional file 7: Table S6. Bodyweight at birth in first generation. All data are expressed as median [Q1;Q3]. (PPTX 39 kb)

Additional file 8: Table S7. Morphological analysis of labyrinthine area at 28 dpc from first generation. All data are expressed as median [Q1;Q3]. (PPTX 63 kb)

Additional file 9: Figure S2. Head measurements during gestation and post-mortem. During gestation, head length was measrued by ultrasound. Post-motem, head measurements were performed using a digital caliper. (PPTX 775 kb)

Additional file 10: Figure S3. Distribution of nanoparticles in the maternal lungs at 28 dpc. Ultrathin sections (75 nm) were obtained from various lung areas: rostral or caudal parenchyma (**a, c, f, g**); rostral or caudal bronchus (**b, d, e, h**). Arrowhaeds indicate particles in alveoli, small arrowheads indicate NP and arrows thin or not indicate isolated particles. Scale bars: (**a**): 20 μm, insert: 10 μm; (**b**): 2 μm; (**c**): 600 nm; (**d**): 1 μm; (**e**): 500 nm; (**f**): 160 nm; (**g**): 700 nm; (**h**): 500 nm, insert: 400 nm. Abbreviations: Br: brochiolus; E: erythrocyte; EC: endothelial cell; Ly: lysosome; M: macrophage; MC: Smooth muscle cell; N: nucleus; NP: nanoparticles; PnI: type I pneumocyte. (PPTX 1172 kb)

Additional file 11: Table S8. Fetoplacental biometry at 28 dpc for the second generation. Female rabbits inhaled 1mg/m3 of NPs, 2 hours/day, 5 days/week, from 3 dpc to 27dpc. Dams were allowed to give birth generation F1. Adult F1 female (7.5 months of age) were mated and euthanized pregnant at 28 dpc. Fetoplacental units of generation F2 were collected in control (C) and exposed (E) groups. Effect of grand-dam (F0) pregnancy exposure to engine diesel exhaust on second-generation fetuses was estimated using linear model with random effect of dam (F1) adjusted for number of fetuses by dam, fetus position in the horn and fetus sex. All data are expressed as median [Q1;Q3]. (*p < 0.05). (PPTX 76 kb)
