# Supplementary figures and images for: Maternal exposure to diluted diesel engine exhaust alters placental function and induces intergenerational effects in rabbits
Source: Part Fibre Toxicol. 2016 Jul 26;13:39. doi: 10.1186/s12989-016-0151-7 (PMC4962477; doi:10.1186/s12989-016-0151-7)

## Slide 1
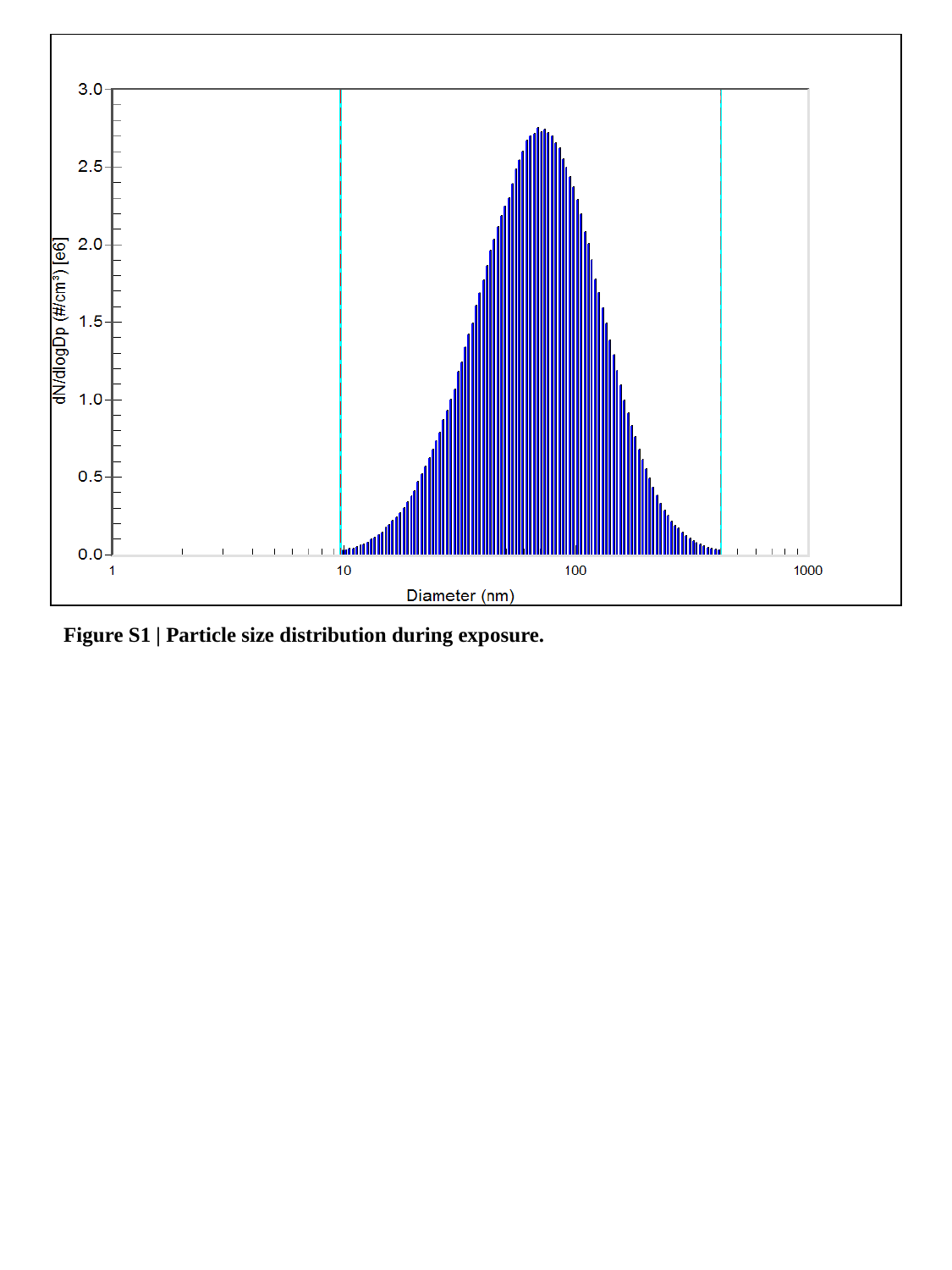

Figure S1 | Particle size distribution during exposure.

Supplement: Supplementary file 1 — Particle size distribution during exposure. (PPTX 52 kb) [file 12989_2016_151_MOESM1_ESM.pptx]
