# Supplementary material for: Maternal exposure to diluted diesel engine exhaust alters placental function and induces intergenerational effects in rabbits
Source: Part Fibre Toxicol. 2016 Jul 26;13:39. doi: 10.1186/s12989-016-0151-7 (PMC4962477; doi:10.1186/s12989-016-0151-7)
Supplement: Supplementary file 2 — Diesel exhaust composition during exposure. NO: Nitrogen Oxid, CO: Carbon Oxid. (PPTX 46 kb) [file 12989_2016_151_MOESM2_ESM.pptx]

## Slide 1
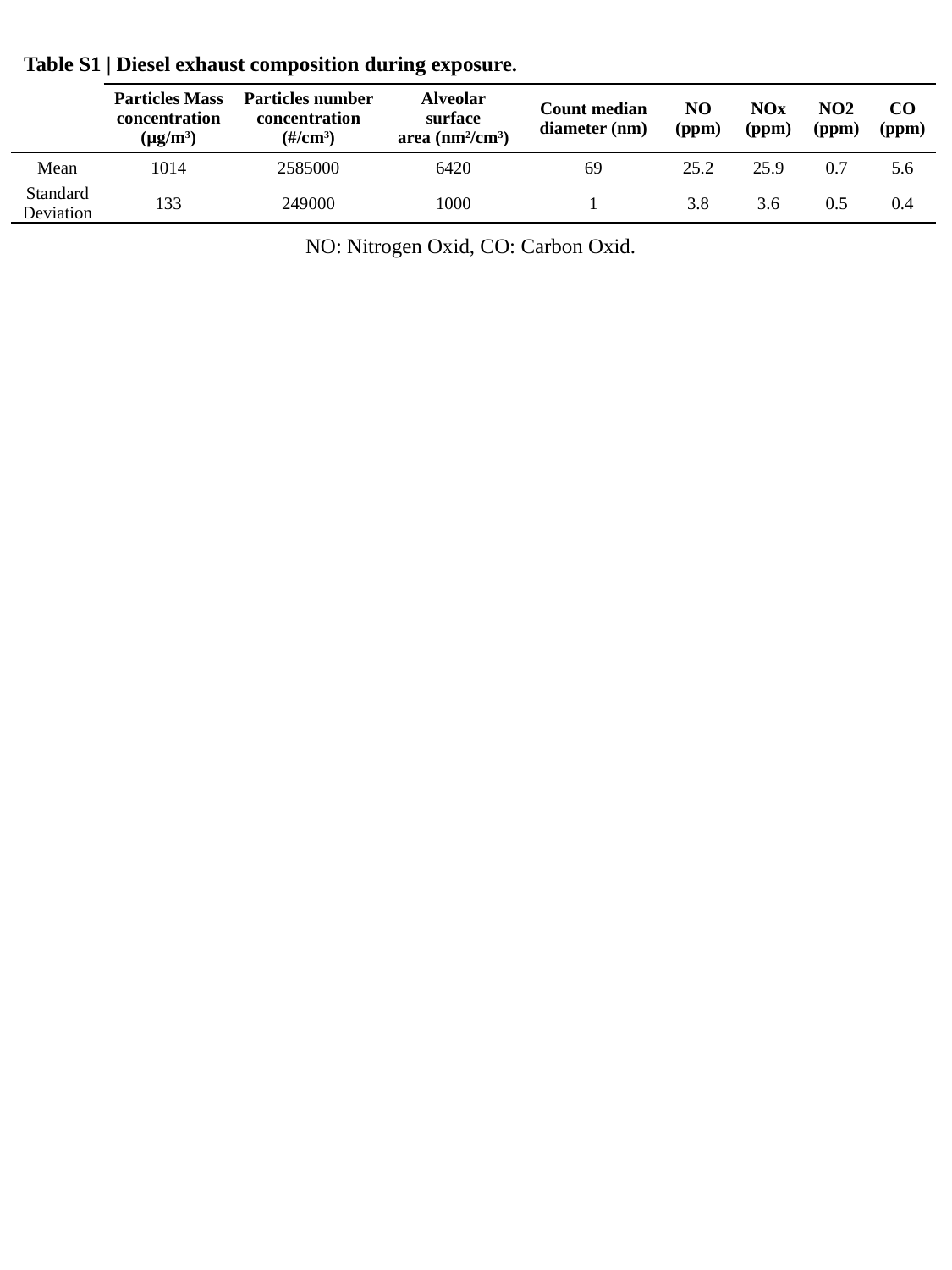

Table S1 | Diesel exhaust composition during exposure.
| | Particles Mass concentration (µg/m3) | Particles number concentration (#/cm3) | Alveolar surface area (nm2/cm3) | Count median diameter (nm) | NO (ppm) | NOx (ppm) | NO2 (ppm) | CO (ppm) |
| --- | --- | --- | --- | --- | --- | --- | --- | --- |
| Mean | 1014 | 2585000 | 6420 | 69 | 25.2 | 25.9 | 0.7 | 5.6 |
| Standard Deviation | 133 | 249000 | 1000 | 1 | 3.8 | 3.6 | 0.5 | 0.4 |
NO: Nitrogen Oxid, CO: Carbon Oxid.
