# Supplementary material for: Maternal exposure to diluted diesel engine exhaust alters placental function and induces intergenerational effects in rabbits
Source: Part Fibre Toxicol. 2016 Jul 26;13:39. doi: 10.1186/s12989-016-0151-7 (PMC4962477; doi:10.1186/s12989-016-0151-7)
Supplement: Supplementary file 3 — Ultrasound embryo measurements at 7 dpc in first generation. Ellipse was used to measure embryo diameters. Diameter 1 represents the longest one and Diameter 2 the shortest one. All data are expressed as median [Q1;Q3]. (PPTX 51 kb) [file 12989_2016_151_MOESM3_ESM.pptx]

## Slide 1
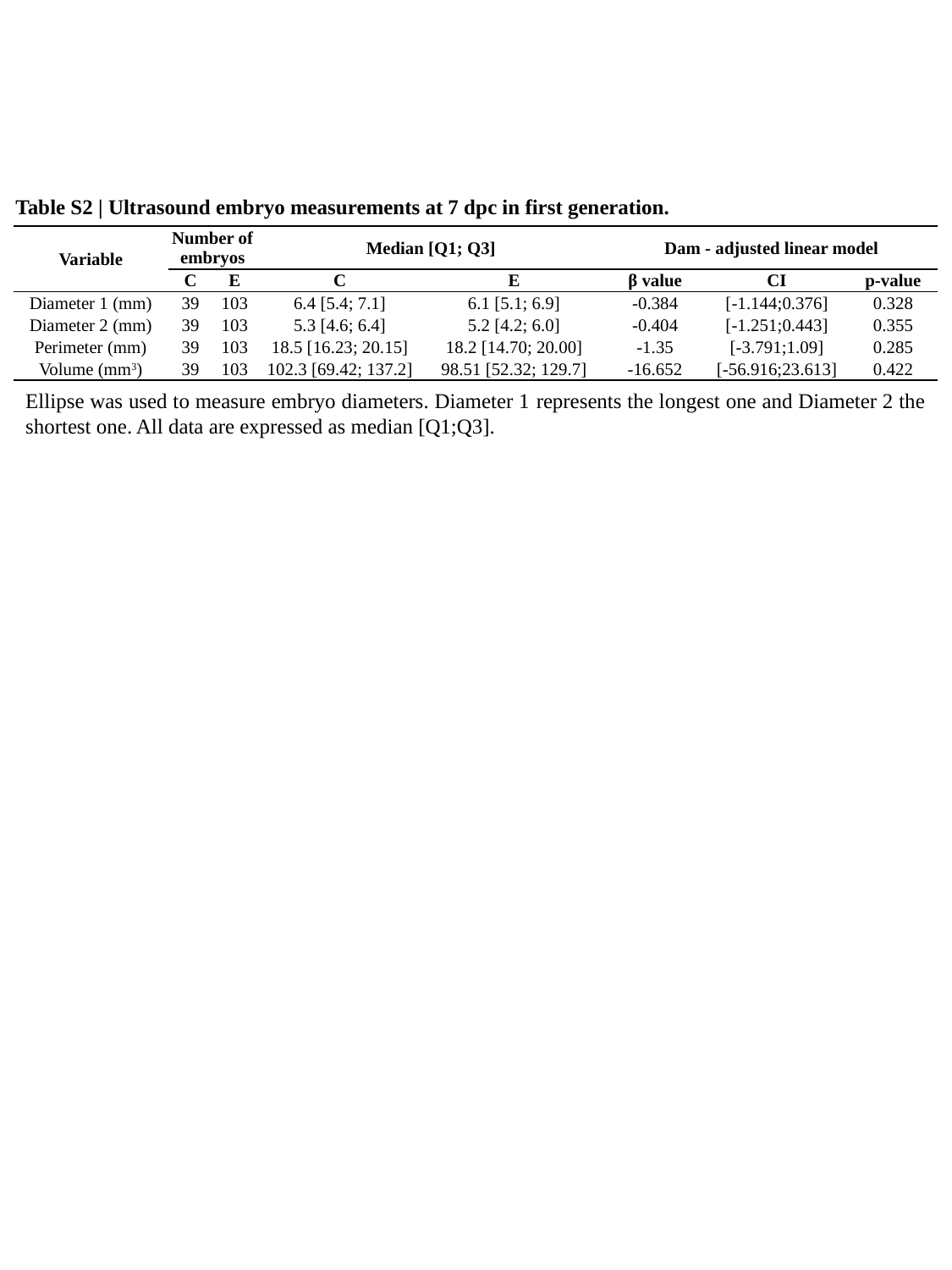

Table S2 | Ultrasound embryo measurements at 7 dpc in first generation.
| Variable | Number of embryos | | Median [Q1; Q3] | | Dam - adjusted linear model | | |
| --- | --- | --- | --- | --- | --- | --- | --- |
| | C | E | C | E | β value | CI | p-value |
| Diameter 1 (mm) | 39 | 103 | 6.4 [5.4; 7.1] | 6.1 [5.1; 6.9] | -0.384 | [-1.144;0.376] | 0.328 |
| Diameter 2 (mm) | 39 | 103 | 5.3 [4.6; 6.4] | 5.2 [4.2; 6.0] | -0.404 | [-1.251;0.443] | 0.355 |
| Perimeter (mm) | 39 | 103 | 18.5 [16.23; 20.15] | 18.2 [14.70; 20.00] | -1.35 | [-3.791;1.09] | 0.285 |
| Volume (mm3) | 39 | 103 | 102.3 [69.42; 137.2] | 98.51 [52.32; 129.7] | -16.652 | [-56.916;23.613] | 0.422 |
Ellipse was used to measure embryo diameters. Diameter 1 represents the longest one and Diameter 2 the shortest one. All data are expressed as median [Q1;Q3].
