# Supplementary material for: Maternal exposure to diluted diesel engine exhaust alters placental function and induces intergenerational effects in rabbits
Source: Part Fibre Toxicol. 2016 Jul 26;13:39. doi: 10.1186/s12989-016-0151-7 (PMC4962477; doi:10.1186/s12989-016-0151-7)
Supplement: Supplementary file 4 — Ultrasound fetal measurements at 21 dpc in the first generation. All data are expressed as median [Q1;Q3]. (PPTX 63 kb) [file 12989_2016_151_MOESM4_ESM.pptx]

## Slide 1
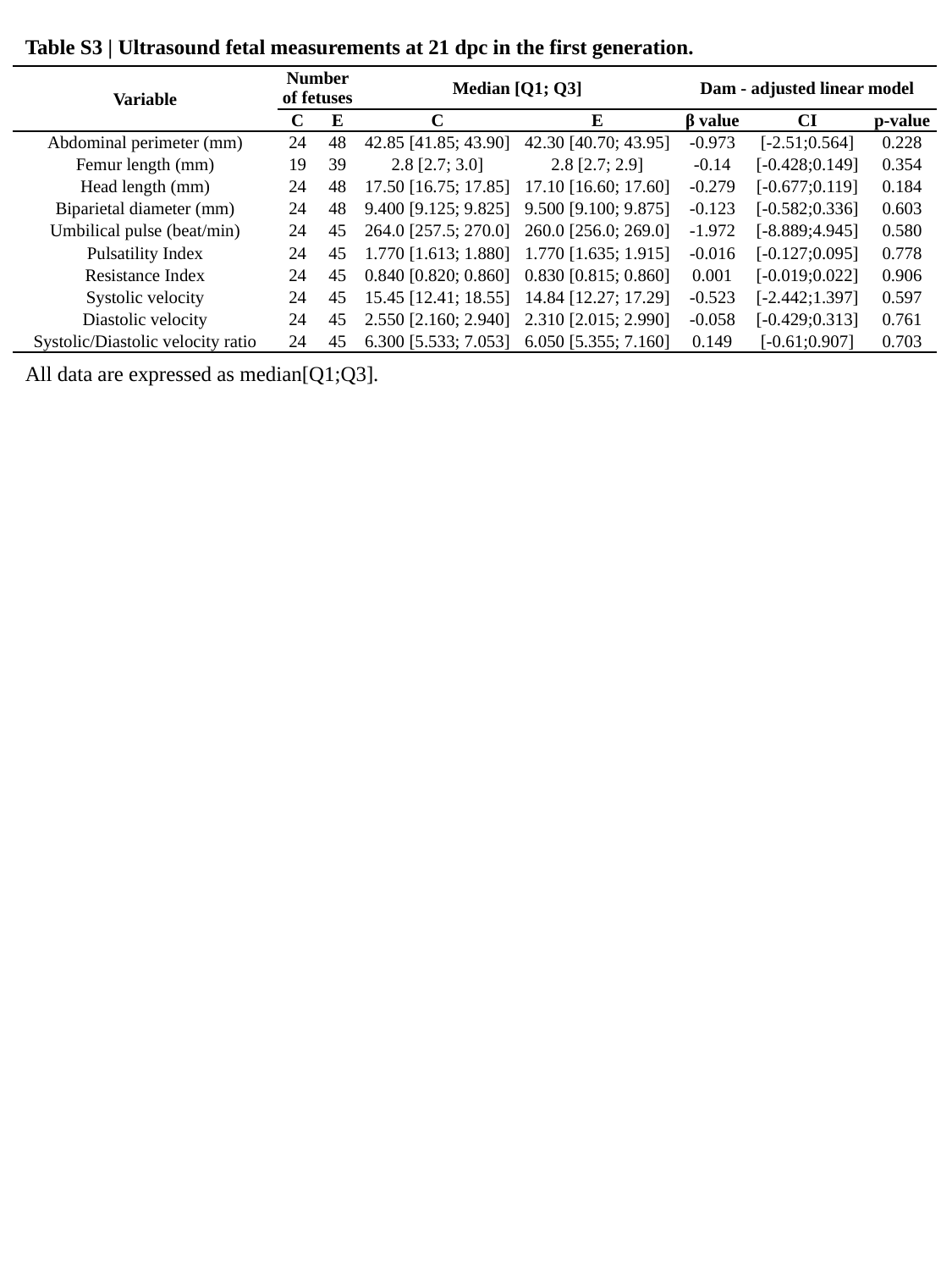

Table S3 | Ultrasound fetal measurements at 21 dpc in the first generation.
| Variable | Number of fetuses | | Median [Q1; Q3] | | Dam - adjusted linear model | | |
| --- | --- | --- | --- | --- | --- | --- | --- |
| | C | E | C | E | β value | CI | p-value |
| Abdominal perimeter (mm) | 24 | 48 | 42.85 [41.85; 43.90] | 42.30 [40.70; 43.95] | -0.973 | [-2.51;0.564] | 0.228 |
| Femur length (mm) | 19 | 39 | 2.8 [2.7; 3.0] | 2.8 [2.7; 2.9] | -0.14 | [-0.428;0.149] | 0.354 |
| Head length (mm) | 24 | 48 | 17.50 [16.75; 17.85] | 17.10 [16.60; 17.60] | -0.279 | [-0.677;0.119] | 0.184 |
| Biparietal diameter (mm) | 24 | 48 | 9.400 [9.125; 9.825] | 9.500 [9.100; 9.875] | -0.123 | [-0.582;0.336] | 0.603 |
| Umbilical pulse (beat/min) | 24 | 45 | 264.0 [257.5; 270.0] | 260.0 [256.0; 269.0] | -1.972 | [-8.889;4.945] | 0.580 |
| Pulsatility Index | 24 | 45 | 1.770 [1.613; 1.880] | 1.770 [1.635; 1.915] | -0.016 | [-0.127;0.095] | 0.778 |
| Resistance Index | 24 | 45 | 0.840 [0.820; 0.860] | 0.830 [0.815; 0.860] | 0.001 | [-0.019;0.022] | 0.906 |
| Systolic velocity | 24 | 45 | 15.45 [12.41; 18.55] | 14.84 [12.27; 17.29] | -0.523 | [-2.442;1.397] | 0.597 |
| Diastolic velocity | 24 | 45 | 2.550 [2.160; 2.940] | 2.310 [2.015; 2.990] | -0.058 | [-0.429;0.313] | 0.761 |
| Systolic/Diastolic velocity ratio | 24 | 45 | 6.300 [5.533; 7.053] | 6.050 [5.355; 7.160] | 0.149 | [-0.61;0.907] | 0.703 |
All data are expressed as median[Q1;Q3].
