# Supplementary material for: Maternal exposure to diluted diesel engine exhaust alters placental function and induces intergenerational effects in rabbits
Source: Part Fibre Toxicol. 2016 Jul 26;13:39. doi: 10.1186/s12989-016-0151-7 (PMC4962477; doi:10.1186/s12989-016-0151-7)
Supplement: Supplementary file 5 — Ultrasound placental measurements at 21 dpc. Mean Grey represents the density of tissu of interest. All data are expressed as median [Q1;Q3]. (PPTX 46 kb) [file 12989_2016_151_MOESM5_ESM.pptx]

## Slide 1
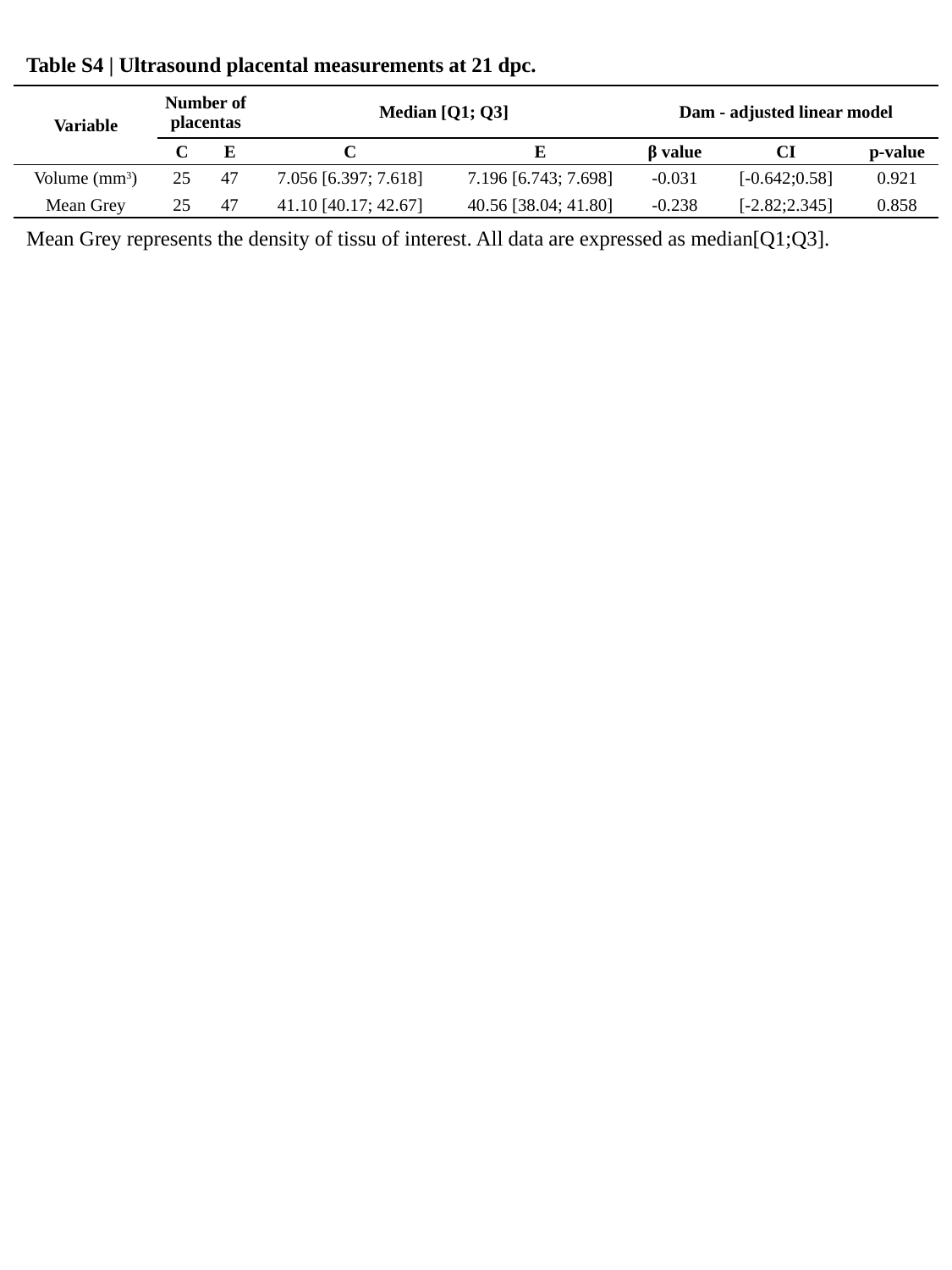

Table S4 | Ultrasound placental measurements at 21 dpc.
| Variable | Number of placentas | | Median [Q1; Q3] | | Dam - adjusted linear model | | |
| --- | --- | --- | --- | --- | --- | --- | --- |
| | C | E | C | E | β value | CI | p-value |
| Volume (mm3) | 25 | 47 | 7.056 [6.397; 7.618] | 7.196 [6.743; 7.698] | -0.031 | [-0.642;0.58] | 0.921 |
| Mean Grey | 25 | 47 | 41.10 [40.17; 42.67] | 40.56 [38.04; 41.80] | -0.238 | [-2.82;2.345] | 0.858 |
Mean Grey represents the density of tissu of interest. All data are expressed as median[Q1;Q3].
