# Supplementary material for: Maternal exposure to diluted diesel engine exhaust alters placental function and induces intergenerational effects in rabbits
Source: Part Fibre Toxicol. 2016 Jul 26;13:39. doi: 10.1186/s12989-016-0151-7 (PMC4962477; doi:10.1186/s12989-016-0151-7)
Supplement: Supplementary file 6 — Ultrasound fetal measurements at 28 dpc. All data are expressed as median [Q1;Q3]. (PPTX 64 kb) [file 12989_2016_151_MOESM6_ESM.pptx]

## Slide 1
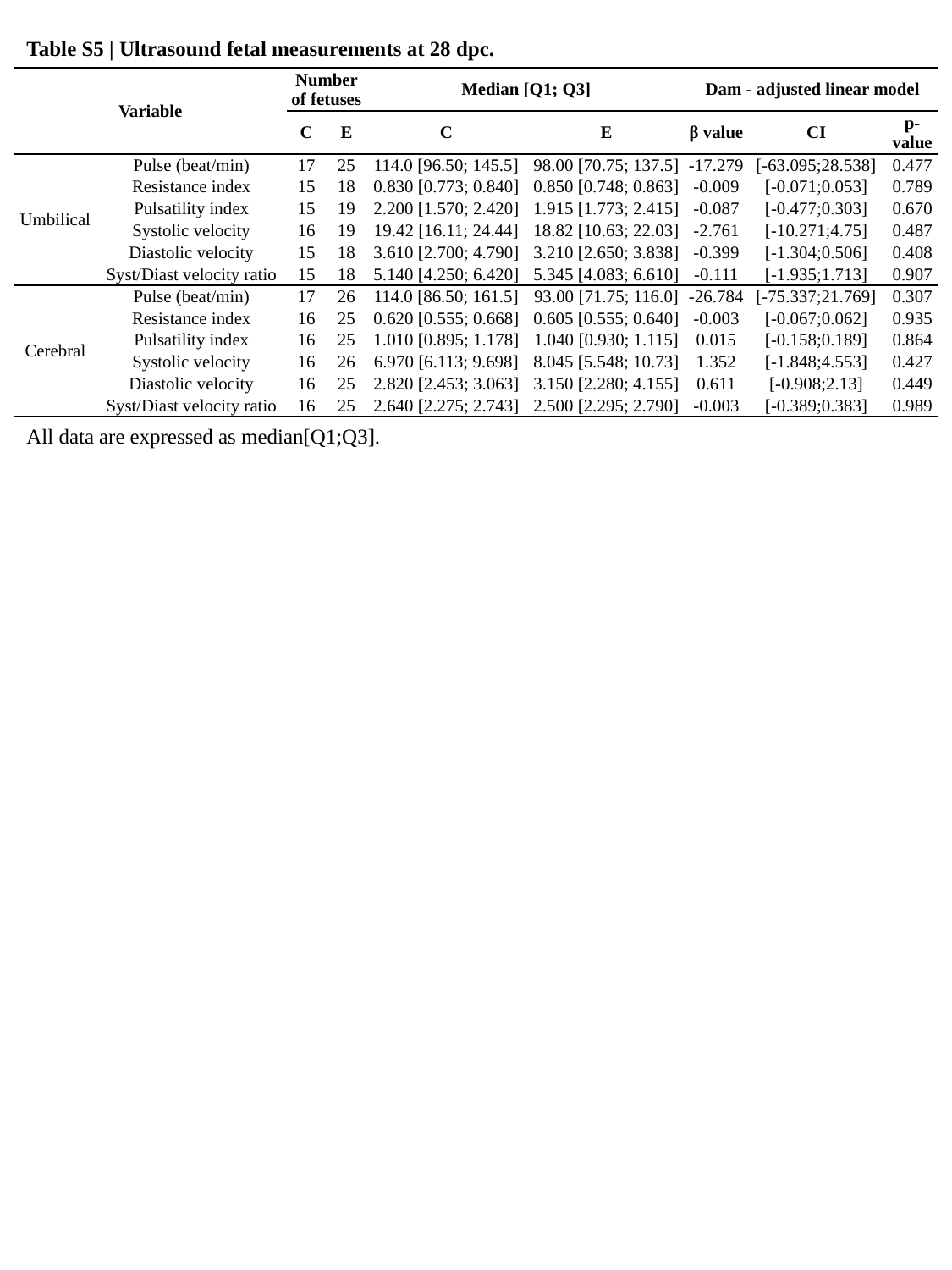

Table S5 | Ultrasound fetal measurements at 28 dpc.
| Variable | | Number of fetuses | | Median [Q1; Q3] | | Dam - adjusted linear model | | |
| --- | --- | --- | --- | --- | --- | --- | --- | --- |
| | | C | E | C | E | β value | CI | p-value |
| Umbilical | Pulse (beat/min) | 17 | 25 | 114.0 [96.50; 145.5] | 98.00 [70.75; 137.5] | -17.279 | [-63.095;28.538] | 0.477 |
| | Resistance index | 15 | 18 | 0.830 [0.773; 0.840] | 0.850 [0.748; 0.863] | -0.009 | [-0.071;0.053] | 0.789 |
| | Pulsatility index | 15 | 19 | 2.200 [1.570; 2.420] | 1.915 [1.773; 2.415] | -0.087 | [-0.477;0.303] | 0.670 |
| | Systolic velocity | 16 | 19 | 19.42 [16.11; 24.44] | 18.82 [10.63; 22.03] | -2.761 | [-10.271;4.75] | 0.487 |
| | Diastolic velocity | 15 | 18 | 3.610 [2.700; 4.790] | 3.210 [2.650; 3.838] | -0.399 | [-1.304;0.506] | 0.408 |
| | Syst/Diast velocity ratio | 15 | 18 | 5.140 [4.250; 6.420] | 5.345 [4.083; 6.610] | -0.111 | [-1.935;1.713] | 0.907 |
| Cerebral | Pulse (beat/min) | 17 | 26 | 114.0 [86.50; 161.5] | 93.00 [71.75; 116.0] | -26.784 | [-75.337;21.769] | 0.307 |
| | Resistance index | 16 | 25 | 0.620 [0.555; 0.668] | 0.605 [0.555; 0.640] | -0.003 | [-0.067;0.062] | 0.935 |
| | Pulsatility index | 16 | 25 | 1.010 [0.895; 1.178] | 1.040 [0.930; 1.115] | 0.015 | [-0.158;0.189] | 0.864 |
| | Systolic velocity | 16 | 26 | 6.970 [6.113; 9.698] | 8.045 [5.548; 10.73] | 1.352 | [-1.848;4.553] | 0.427 |
| | Diastolic velocity | 16 | 25 | 2.820 [2.453; 3.063] | 3.150 [2.280; 4.155] | 0.611 | [-0.908;2.13] | 0.449 |
| | Syst/Diast velocity ratio | 16 | 25 | 2.640 [2.275; 2.743] | 2.500 [2.295; 2.790] | -0.003 | [-0.389;0.383] | 0.989 |
All data are expressed as median[Q1;Q3].
