# Supplementary material for: Maternal exposure to diluted diesel engine exhaust alters placental function and induces intergenerational effects in rabbits
Source: Part Fibre Toxicol. 2016 Jul 26;13:39. doi: 10.1186/s12989-016-0151-7 (PMC4962477; doi:10.1186/s12989-016-0151-7)
Supplement: Supplementary file 7 — Bodyweight at birth in first generation. All data are expressed as median [Q1;Q3]. (PPTX 39 kb) [file 12989_2016_151_MOESM7_ESM.pptx]

## Slide 1
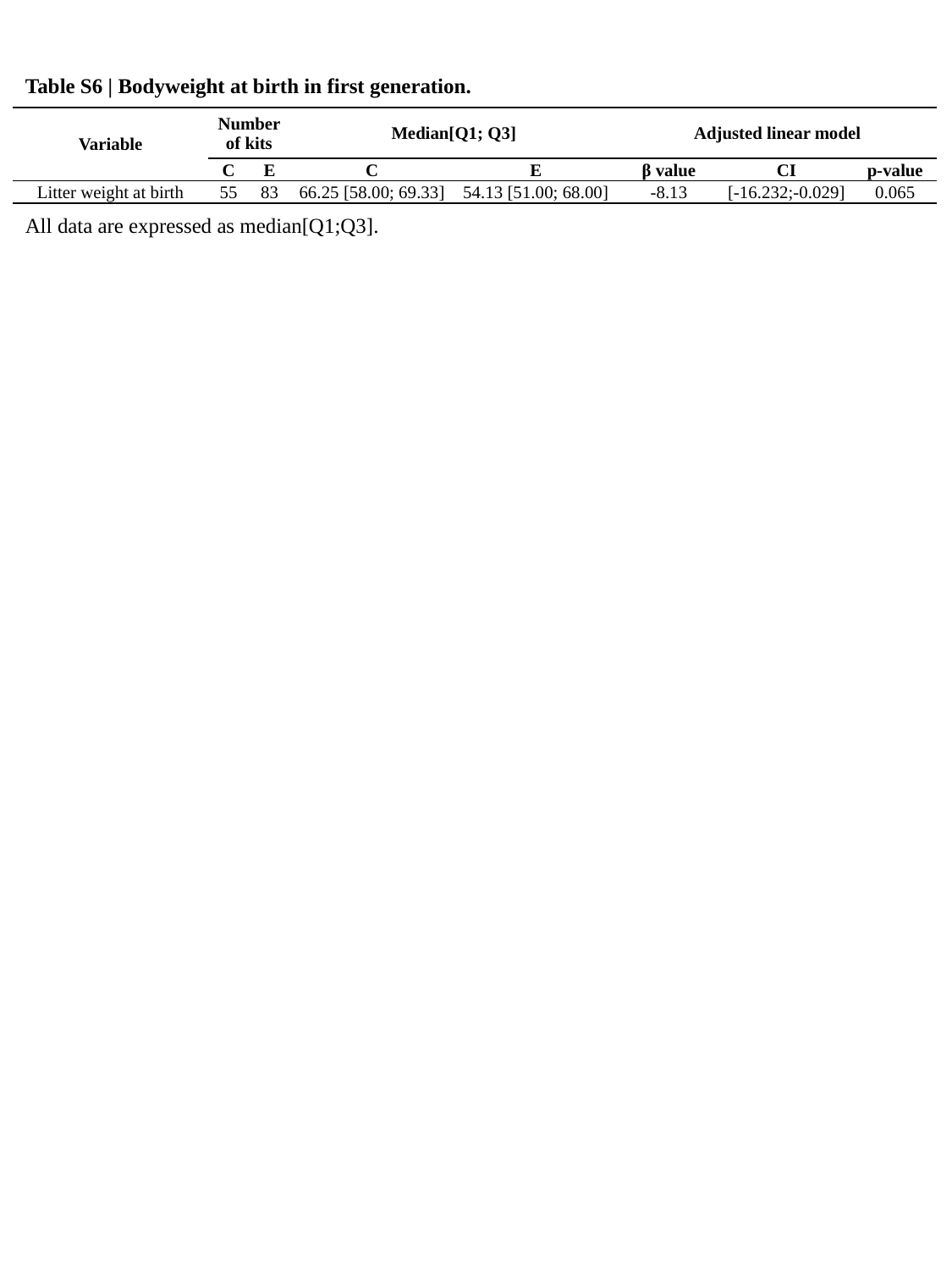

Table S6 | Bodyweight at birth in first generation.
| Variable | Number of kits | | Median[Q1; Q3] | | Adjusted linear model | | |
| --- | --- | --- | --- | --- | --- | --- | --- |
| | C | E | C | E | β value | CI | p-value |
| Litter weight at birth | 55 | 83 | 66.25 [58.00; 69.33] | 54.13 [51.00; 68.00] | -8.13 | [-16.232;-0.029] | 0.065 |
All data are expressed as median[Q1;Q3].
