# Supplementary material for: Maternal exposure to diluted diesel engine exhaust alters placental function and induces intergenerational effects in rabbits
Source: Part Fibre Toxicol. 2016 Jul 26;13:39. doi: 10.1186/s12989-016-0151-7 (PMC4962477; doi:10.1186/s12989-016-0151-7)
Supplement: Supplementary file 8 — Morphological analysis of labyrinthine area at 28 dpc from first generation. All data are expressed as median [Q1;Q3]. (PPTX 63 kb) [file 12989_2016_151_MOESM8_ESM.pptx]

## Slide 1
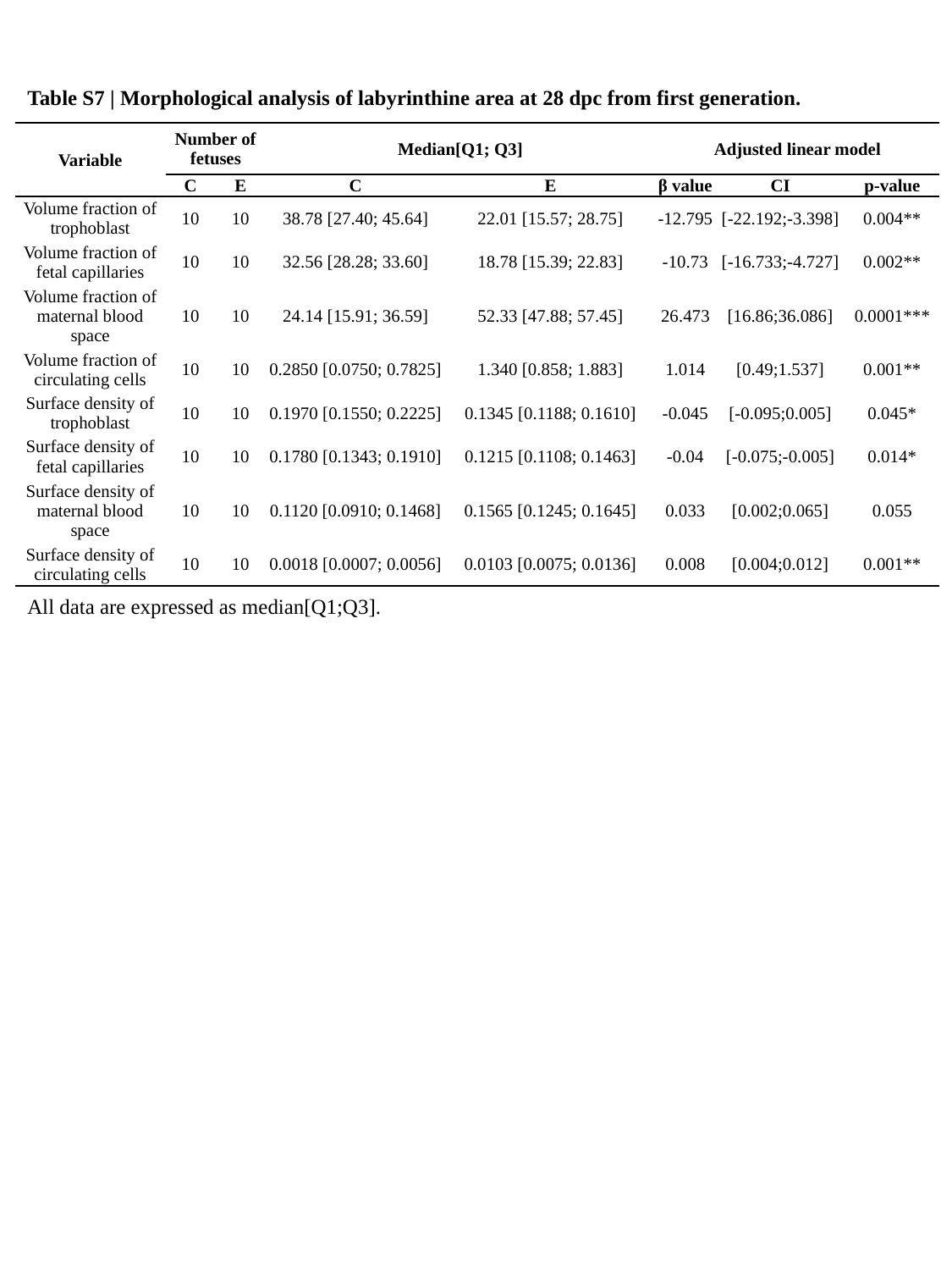

Table S7 | Morphological analysis of labyrinthine area at 28 dpc from first generation.
| Variable | Number of fetuses | | Median[Q1; Q3] | | Adjusted linear model | | |
| --- | --- | --- | --- | --- | --- | --- | --- |
| | C | E | C | E | β value | CI | p-value |
| Volume fraction of trophoblast | 10 | 10 | 38.78 [27.40; 45.64] | 22.01 [15.57; 28.75] | -12.795 | [-22.192;-3.398] | 0.004\*\* |
| Volume fraction of fetal capillaries | 10 | 10 | 32.56 [28.28; 33.60] | 18.78 [15.39; 22.83] | -10.73 | [-16.733;-4.727] | 0.002\*\* |
| Volume fraction of maternal blood space | 10 | 10 | 24.14 [15.91; 36.59] | 52.33 [47.88; 57.45] | 26.473 | [16.86;36.086] | 0.0001\*\*\* |
| Volume fraction of circulating cells | 10 | 10 | 0.2850 [0.0750; 0.7825] | 1.340 [0.858; 1.883] | 1.014 | [0.49;1.537] | 0.001\*\* |
| Surface density of trophoblast | 10 | 10 | 0.1970 [0.1550; 0.2225] | 0.1345 [0.1188; 0.1610] | -0.045 | [-0.095;0.005] | 0.045\* |
| Surface density of fetal capillaries | 10 | 10 | 0.1780 [0.1343; 0.1910] | 0.1215 [0.1108; 0.1463] | -0.04 | [-0.075;-0.005] | 0.014\* |
| Surface density of maternal blood space | 10 | 10 | 0.1120 [0.0910; 0.1468] | 0.1565 [0.1245; 0.1645] | 0.033 | [0.002;0.065] | 0.055 |
| Surface density of circulating cells | 10 | 10 | 0.0018 [0.0007; 0.0056] | 0.0103 [0.0075; 0.0136] | 0.008 | [0.004;0.012] | 0.001\*\* |
All data are expressed as median[Q1;Q3].
