# Supplementary material for: Maternal exposure to diluted diesel engine exhaust alters placental function and induces intergenerational effects in rabbits
Source: Part Fibre Toxicol. 2016 Jul 26;13:39. doi: 10.1186/s12989-016-0151-7 (PMC4962477; doi:10.1186/s12989-016-0151-7)
Supplement: Supplementary file 9 — Head measurements during gestation and post-mortem. During gestation, head length was measrued by ultrasound. Post-motem, head measurements were performed using a digital caliper. (PPTX 775 kb) [file 12989_2016_151_MOESM9_ESM.pptx]

## Slide 1
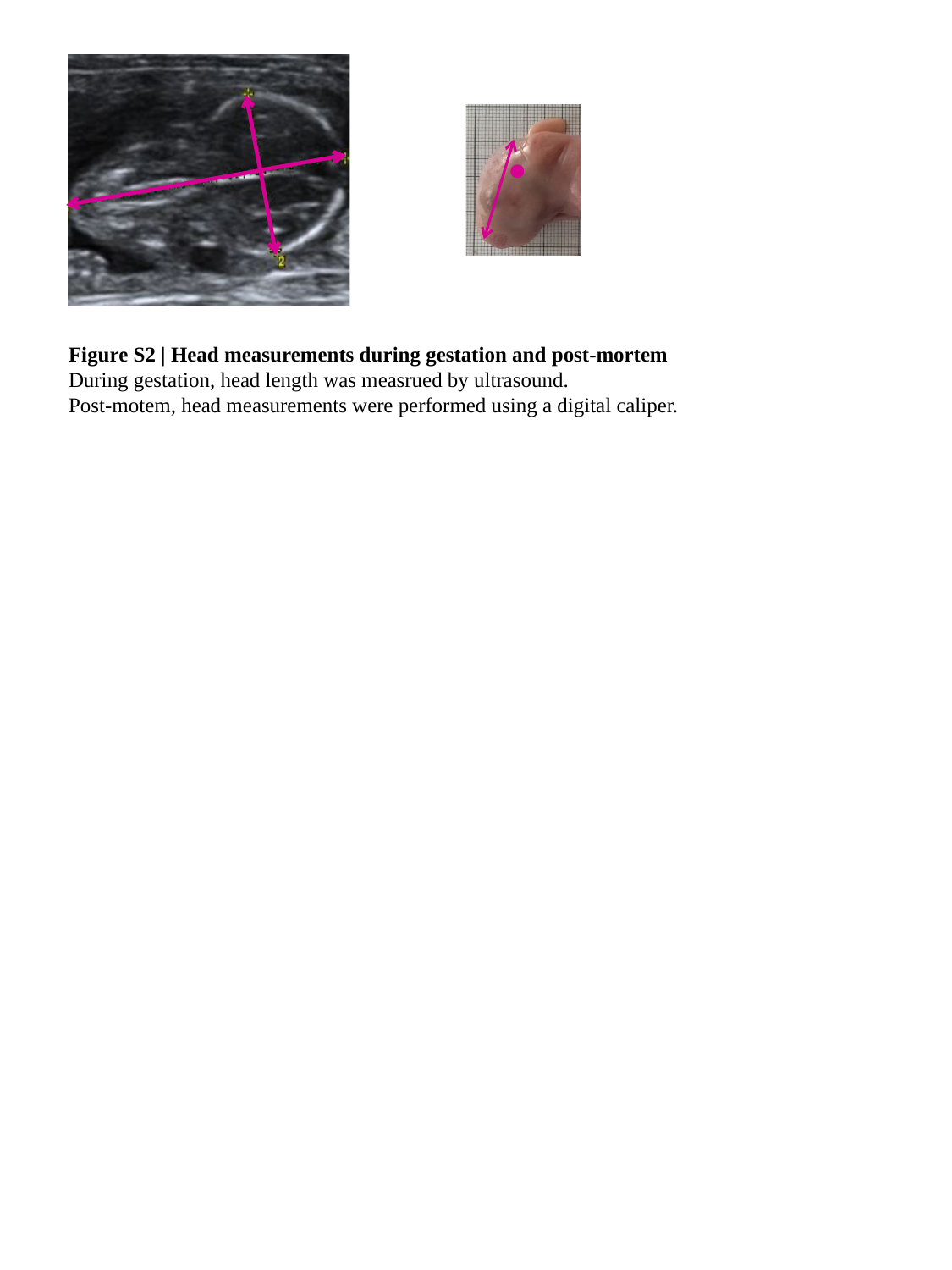

Figure S2 | Head measurements during gestation and post-mortem
During gestation, head length was measrued by ultrasound.
Post-motem, head measurements were performed using a digital caliper.
