# Supplementary material for: Maternal exposure to diluted diesel engine exhaust alters placental function and induces intergenerational effects in rabbits
Source: Part Fibre Toxicol. 2016 Jul 26;13:39. doi: 10.1186/s12989-016-0151-7 (PMC4962477; doi:10.1186/s12989-016-0151-7)
Supplement: Supplementary file 10 — Distribution of nanoparticles in the maternal lungs at 28 dpc. Ultrathin sections (75 nm) were obtained from various lung areas: rostral or caudal parenchyma (a, c, f, g); rostral or caudal bronchus (b, d, e, h). Arrowhaeds indicate particles in alveoli, small arrowheads indicate NP and arrows thin or not indicate isolated particles. Scale bars: (a): 20 μm, insert: 10 μm; (b): 2 μm; (c): 600 nm; (d): 1 μm; (e): 500 nm; (f): 160 nm; (g): 700 nm; (h): 500 nm, insert: 400 nm. Abbreviations: Br: brochiolus; E: erythrocyte; EC: endothelial cell; Ly: lysosome; M: macrophage; MC: Smooth muscle cell; N: nucleus; NP: nanoparticles; PnI: type I pneumocyte. (PPTX 1172 kb) [file 12989_2016_151_MOESM10_ESM.pptx]

## Slide 1
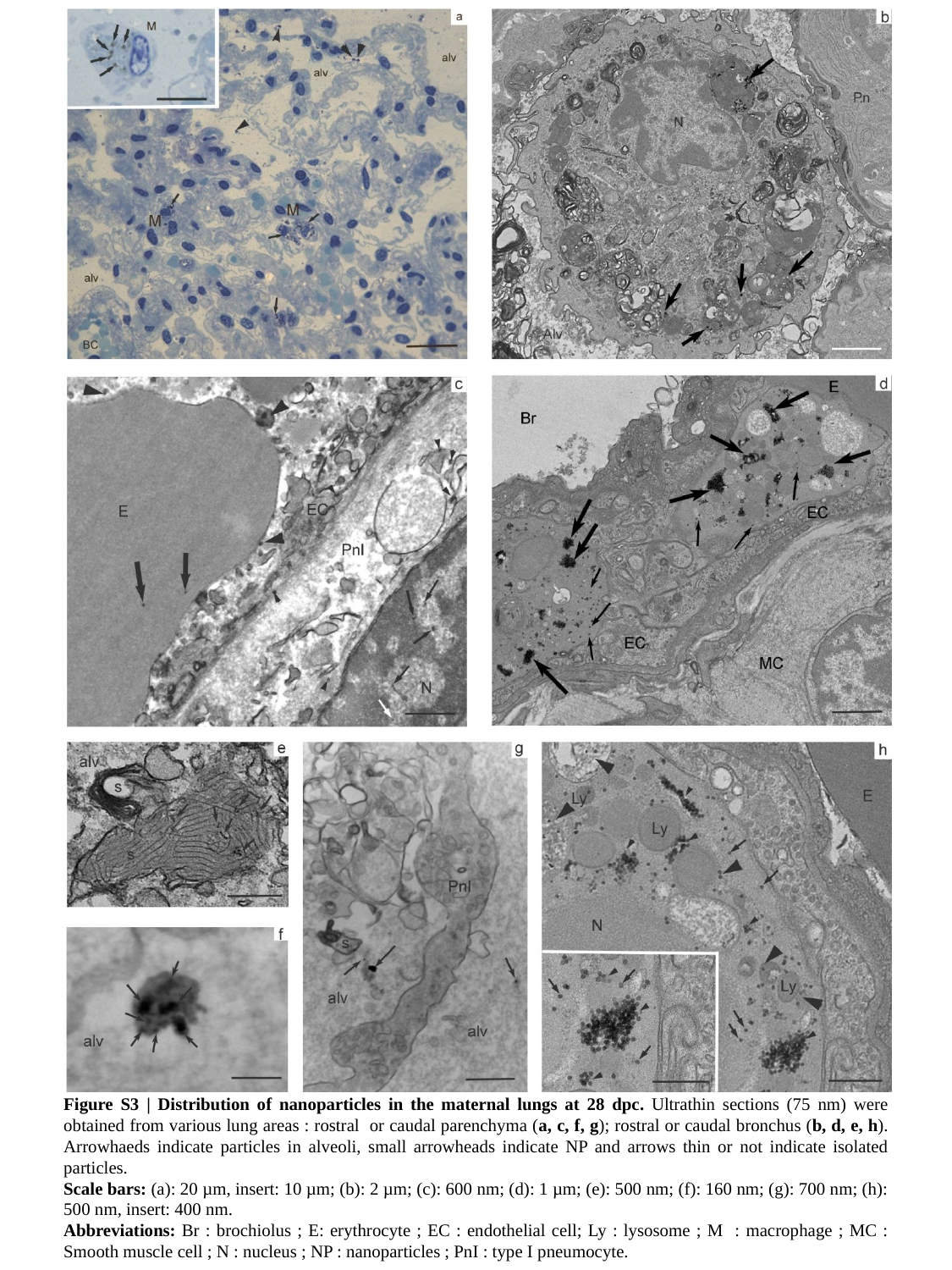

Figure S3 | Distribution of nanoparticles in the maternal lungs at 28 dpc. Ultrathin sections (75 nm) were obtained from various lung areas : rostral or caudal parenchyma (a, c, f, g); rostral or caudal bronchus (b, d, e, h). Arrowhaeds indicate particles in alveoli, small arrowheads indicate NP and arrows thin or not indicate isolated particles.
Scale bars: (a): 20 µm, insert: 10 µm; (b): 2 µm; (c): 600 nm; (d): 1 µm; (e): 500 nm; (f): 160 nm; (g): 700 nm; (h): 500 nm, insert: 400 nm.
Abbreviations: Br : brochiolus ; E: erythrocyte ; EC : endothelial cell; Ly : lysosome ; M : macrophage ; MC : Smooth muscle cell ; N : nucleus ; NP : nanoparticles ; PnI : type I pneumocyte.
