# Supplementary material for: Maternal exposure to diluted diesel engine exhaust alters placental function and induces intergenerational effects in rabbits
Source: Part Fibre Toxicol. 2016 Jul 26;13:39. doi: 10.1186/s12989-016-0151-7 (PMC4962477; doi:10.1186/s12989-016-0151-7)
Supplement: Supplementary file 11 — Fetoplacental biometry at 28 dpc for the second generation. Female rabbits inhaled 1mg/m3 of NPs, 2 hours/day, 5 days/week, from 3 dpc to 27dpc. Dams were allowed to give birth generation F1. Adult F1 female (7.5 months of age) were mated and euthanized pregnant at 28 dpc. Fetoplacental units of generation F2 were collected in control (C) and exposed (E) groups. Effect of grand-dam (F0) pregnancy exposure to engine diesel exhaust on second-generation fetuses was estimated using linear model with random effect of dam (F1) adjusted for number of fetuses by dam, fetus position in the horn and fetus sex. All data are expressed as median [Q1;Q3]. (*p < 0.05). (PPTX 76 kb) [file 12989_2016_151_MOESM11_ESM.pptx]

## Slide 1
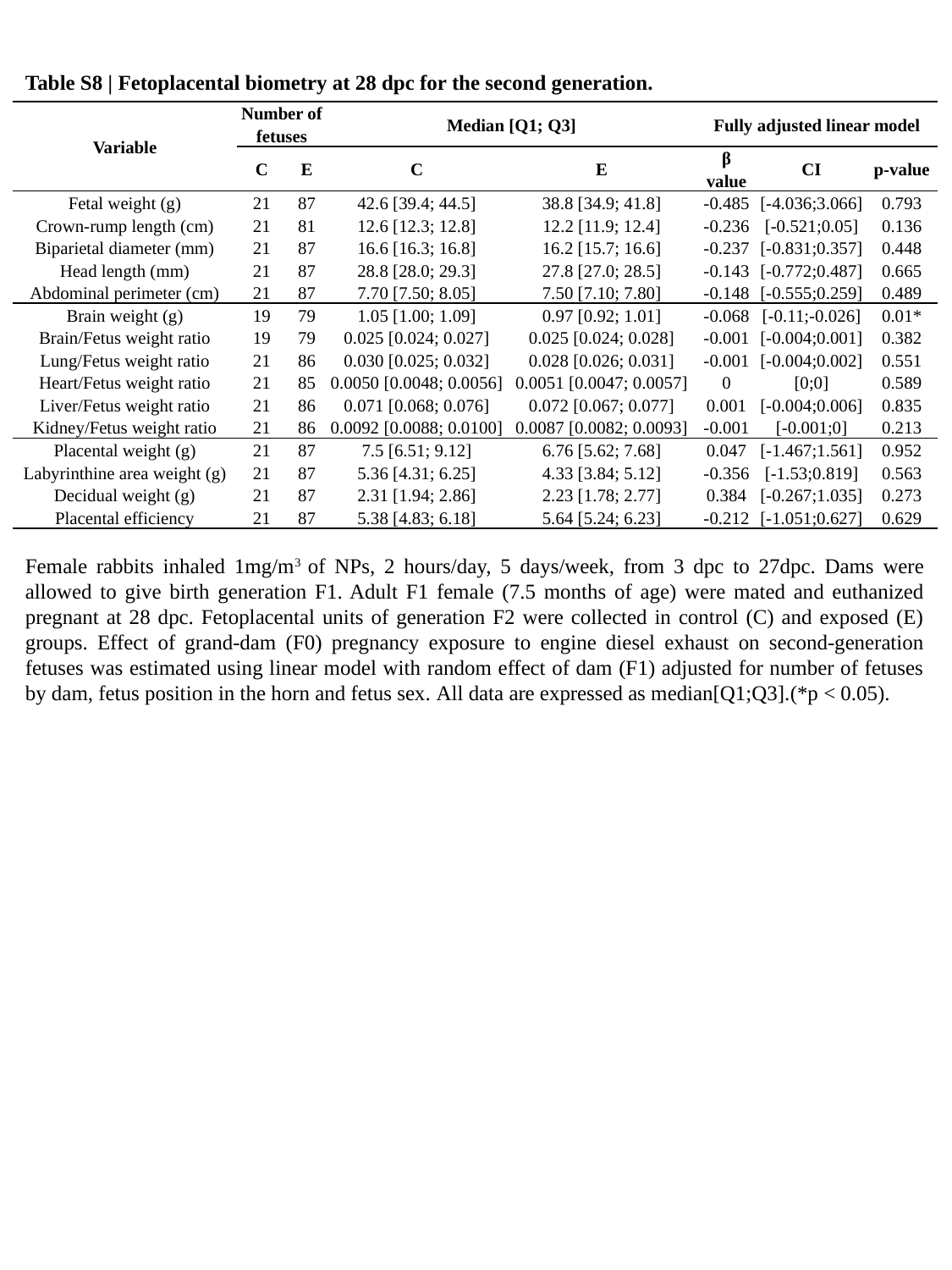

Table S8 | Fetoplacental biometry at 28 dpc for the second generation.
| Variable | Number of fetuses | | Median [Q1; Q3] | | Fully adjusted linear model | | |
| --- | --- | --- | --- | --- | --- | --- | --- |
| | C | E | C | E | β value | CI | p-value |
| Fetal weight (g) | 21 | 87 | 42.6 [39.4; 44.5] | 38.8 [34.9; 41.8] | -0.485 | [-4.036;3.066] | 0.793 |
| Crown-rump length (cm) | 21 | 81 | 12.6 [12.3; 12.8] | 12.2 [11.9; 12.4] | -0.236 | [-0.521;0.05] | 0.136 |
| Biparietal diameter (mm) | 21 | 87 | 16.6 [16.3; 16.8] | 16.2 [15.7; 16.6] | -0.237 | [-0.831;0.357] | 0.448 |
| Head length (mm) | 21 | 87 | 28.8 [28.0; 29.3] | 27.8 [27.0; 28.5] | -0.143 | [-0.772;0.487] | 0.665 |
| Abdominal perimeter (cm) | 21 | 87 | 7.70 [7.50; 8.05] | 7.50 [7.10; 7.80] | -0.148 | [-0.555;0.259] | 0.489 |
| Brain weight (g) | 19 | 79 | 1.05 [1.00; 1.09] | 0.97 [0.92; 1.01] | -0.068 | [-0.11;-0.026] | 0.01\* |
| Brain/Fetus weight ratio | 19 | 79 | 0.025 [0.024; 0.027] | 0.025 [0.024; 0.028] | -0.001 | [-0.004;0.001] | 0.382 |
| Lung/Fetus weight ratio | 21 | 86 | 0.030 [0.025; 0.032] | 0.028 [0.026; 0.031] | -0.001 | [-0.004;0.002] | 0.551 |
| Heart/Fetus weight ratio | 21 | 85 | 0.0050 [0.0048; 0.0056] | 0.0051 [0.0047; 0.0057] | 0 | [0;0] | 0.589 |
| Liver/Fetus weight ratio | 21 | 86 | 0.071 [0.068; 0.076] | 0.072 [0.067; 0.077] | 0.001 | [-0.004;0.006] | 0.835 |
| Kidney/Fetus weight ratio | 21 | 86 | 0.0092 [0.0088; 0.0100] | 0.0087 [0.0082; 0.0093] | -0.001 | [-0.001;0] | 0.213 |
| Placental weight (g) | 21 | 87 | 7.5 [6.51; 9.12] | 6.76 [5.62; 7.68] | 0.047 | [-1.467;1.561] | 0.952 |
| Labyrinthine area weight (g) | 21 | 87 | 5.36 [4.31; 6.25] | 4.33 [3.84; 5.12] | -0.356 | [-1.53;0.819] | 0.563 |
| Decidual weight (g) | 21 | 87 | 2.31 [1.94; 2.86] | 2.23 [1.78; 2.77] | 0.384 | [-0.267;1.035] | 0.273 |
| Placental efficiency | 21 | 87 | 5.38 [4.83; 6.18] | 5.64 [5.24; 6.23] | -0.212 | [-1.051;0.627] | 0.629 |
Female rabbits inhaled 1mg/m3 of NPs, 2 hours/day, 5 days/week, from 3 dpc to 27dpc. Dams were allowed to give birth generation F1. Adult F1 female (7.5 months of age) were mated and euthanized pregnant at 28 dpc. Fetoplacental units of generation F2 were collected in control (C) and exposed (E) groups. Effect of grand-dam (F0) pregnancy exposure to engine diesel exhaust on second-generation fetuses was estimated using linear model with random effect of dam (F1) adjusted for number of fetuses by dam, fetus position in the horn and fetus sex. All data are expressed as median[Q1;Q3].(*p < 0.05).
